# Iron oxide nanoparticles as positive T_1_ contrast agents for low-field magnetic resonance imaging at 64 mT

**DOI:** 10.1038/s41598-023-38222-6

**Published:** 2023-07-17

**Authors:** Samuel D. Oberdick, Kalina V. Jordanova, John T. Lundstrom, Giacomo Parigi, Megan E. Poorman, Gary Zabow, Kathryn E. Keenan

**Affiliations:** 1 Department of Physics, University of Colorado, Boulder, CO 80309 USA; 2 National Institute of Standards and Technology, Boulder, CO 80305 USA; 3 Magnetic Resonance Center (CERM), University of Florence, Via Luigi Sacconi 6, 50019 Sesto Fiorentino, Italy; 4 Department of Chemistry “Ugo Schiff”, University of Florence, Via Della Lastruccia 3, 50019 Sesto Fiorentino, Italy; 5 Consorzio Interuniversitario Risonanze Magnetiche Metallo Proteine (CIRMMP), Via Luigi Sacconi 6, 50019 Sesto Fiorentino, Italy; 6 Hyperfine, Inc., Guilford, CT USA

**Keywords:** Magnetic resonance imaging, Imaging techniques and agents

## Abstract

We have investigated the efficacy of superparamagnetic iron oxide nanoparticles (SPIONs) as positive T_1_ contrast agents for low-field magnetic resonance imaging (MRI) at 64 millitesla (mT). Iron oxide-based agents, such as the FDA-approved ferumoxytol, were measured using a variety of techniques to evaluate T_1_ contrast at 64 mT. Additionally, we characterized monodispersed carboxylic acid-coated SPIONs with a range of diameters (4.9–15.7 nm) in order to understand size-dependent properties of T_1_ contrast at low-field. MRI contrast properties were measured using 64 mT MRI, magnetometry, and nuclear magnetic resonance dispersion (NMRD). We also measured MRI contrast at 3 T to provide comparison to a standard clinical field strength. SPIONs have the capacity to perform well as T_1_ contrast agents at 64 mT, with measured longitudinal relaxivity (r_1_) values of up to 67 L mmol^−1^ s^−1^, more than an order of magnitude higher than corresponding r_1_ values at 3 T. The particles exhibit size-dependent longitudinal relaxivities and outperform a commercial Gd-based agent (gadobenate dimeglumine) by more than eight-fold at physiological temperatures. Additionally, we characterize the ratio of transverse to longitudinal relaxivity, r_2_/r_1_ and find that it is ~ 1 for the SPION based agents at 64 mT, indicating a favorable balance of relaxivities for T_1_-weighted contrast imaging. We also correlate the magnetic and structural properties of the particles with models of nanoparticle relaxivity to understand generation of T_1_ contrast. These experiments show that SPIONs, at low fields being targeted for point-of-care low-field MRI systems, have a unique combination of magnetic and structural properties that produce large T_1_ relaxivities.

## Introduction

Low-field magnetic resonance imaging (MRI) has the potential to revolutionize accessibility of MRI for patient diagnosis and neuroimaging^1–5^. The term “low-field” describes MRI scanners that operate at reduced fields (1–100 mT) compared to standard clinical MRI scanners (1.5–3 T). The lower magnetic fields can be generated using permanent magnets and therefore require less power, space, and accompanying infrastructure than clinical-field scanners that use cryogenic superconducting magnets^6,7^. The signal-to-noise ratio (SNR) of MRI scales approximately quadratically with applied field strength, requiring compromises in resolution or scan time to recover the signal. Recently, however, improvements in hardware and advanced image reconstruction (such as Deep Learning and post processing) have led to development of a new generation of low-field scanners^8,9^. Despite the lower SNR, advancements in both hardware and software have enabled low-field scanners that provide diagnostically relevant information within reasonable scan times, albeit with lower spatial resolution than clinical field strengths. Already, low field MRI scanners have been used to identify neurological pathology associated with strokes, hemorrhage, brain tumor, traumatic brain injury, and COVID-19^10,11^. The pathology is identifiable in T_1_-weighted, T_2_-weighted, T_2_ fluid-attenuated inversion recovery, and diffusion-weighted sequences using intrinsic tissue contrast at low-field. The scanners are highly portable, and, in some cases, the power requirements are low enough that a conventional wall socket can be used as a power source. As a result, low-field scanners have the potential to increase the accessibility of MRI in resource limited areas of the world^12^. Low-field MRI can also open up new avenues for point-of-care medical imaging. For example, low-field MRI can be performed at a patient’s bedside in intensive care units, enabling timely imaging to be performed in critically-ill patients who are too difficult to move^13^. We note that the range of magnetic field strengths below 0.1 T is sometimes referred to as “ultra-low-field” or “very-low-field” to distinguish from other commercial devices operating at intermediate fields^9^. The work described herein focuses on 64 mT and is referred to as “low-field” MRI for simplicity.

In 2020 the US Food and Drug Administration approved a portable low-field scanner operating at 64 mT for neuroimaging (Hyperfine Swoop, Guilford, CT, USA). The 64 mT MRI scanner operates at a field that is nearly 47 × smaller than 3 T clinical MRI. It can be powered via a standard wall socket and does not require cryogens. Portable MRI using these scanners can dramatically change the standard neuroimaging workflow. For instance, 64 mT MRI has been used to evaluate brain injury in intensive care units and perform neuroimaging on highly contagious patients with COVID-19^10^. Low-field MRI at 64 mT has also been used to assess intracerebral hemorrhages^14^, intracranial midline shift in stroke patients^15^, and hypoxic ischemic brain injury after cardiac arrest^16^. As low-field imaging becomes widespread, new imaging procedures specifically suited this magnetic field strength are needed, one of which is the exploration, evaluation, and characterization of contrast-enhanced MRI for low-field applications.

Contrast-enhanced MRI is currently used in about 25% of all MRI examinations^17^ at clinical field strengths. Contrast agents that have been traditionally used for clinical field strengths may be suboptimal in the low field-regime, since MRI contrast generation depends on a complex combination of factors and can change considerably depending on field strength. Therefore, we expect that novel low-field contrast agents will play an emerging role in low-field MRI. Contrast agents are typically categorized as either positive or negative contrast agents, depending on whether they increase or decrease signal, respectively, in MR images. Positive T_1_ contrast agents work by shortening the characteristic longitudinal T_1_ relaxation time, thus creating regions with increased signal. Negative T_2_/T_2_* contrast agents operate by reducing the T_2_ or T_2_* time associated with transverse relaxation and decrease signal. Positive T_1_ contrast agents have an advantage compared to negative contrast agents since they operate via signal increase. T_2_/T_2_* agents can obscure underlying contrast in MR image features from signal reduction. Additionally, by shortening the T_1_ time, it is possible to decrease the repetition time of experiments, allowing for an increased number of scans per unit time. T_1_ agents are also considerably less ambiguous than dark markers, since dark contrast can be caused by a variety of signal reducing mechanisms, such as air/water interfaces (i.e., bubbles) or accumulation of biogenic iron.

Candidates for effective T_1_ contrast agents should have high longitudinal relaxivities, meaning they efficiently produce T_1_ relaxation. High relaxivity is preferable because it means that smaller quantities of contrast agent can be used to create perceptible bright markers on an MR image. Besides high relaxivity, it is important that T_1_ contrast agents have a ratio of transverse relaxivity to longitudinal relaxivity that is on the order of one. That way, transverse relaxivity does not dominate the net relaxation effect produced by a contrast agent.

At clinical field strengths, contrast-enhanced MRI is dominated by the use of Gd-based chelates^17^. However, there have been recent concerns regarding the safety of Gd-based contrast agents, specifically with regards to toxicity and long-term deposition in the brain^18,19^. Iron oxide nanoparticles have been previously explored for contrast-enhanced MRI at clinical field strengths, specifically for imaging of the liver and spleen^20,21^. SPIONs are potentially less toxic than Gd since iron oxides can be metabolized by the body. At clinical fields, though, their high magnetic susceptibility generally favors increased transverse relaxivity over longitudinal relaxivity, so they have largely been thought of as negative contrast agents. Alternatively, SPIONs with “ultra-small” diameters of less than 4 nm can exhibit enhanced positive T_1_-weighted contrast at clinical field strengths^22–26^; however, these particles require substantial expertise to synthesize.

At field strengths below standard clinical imaging fields, SPIONs have shown exciting promise as T_1_ agents. Preliminary in vivo studies have been performed at 64 mT using ferumoxytol, which is an FDA-approved SPION-based treatment of iron deficiency anemia^9,27^. In these studies, patients received ferumoxytol for anemia treatment and were later imaged using 64 mT MRI. Contrast-enhanced cerebral vasculature was observed as a result of the ferumoxytol injections. Ferumoxytol has also been explored for off-label use as a contrast agent at 0.25 T and can generate comparable signal enhancement to a Gd-chelate with a lower net concentration of metal (Fe versus Gd)^28^. At 0.13 mT, the longitudinal relaxivity of SPIONs has been measured to be 615 L mmol^−1^ s^−1^, which is two orders of magnitude larger than Gd-based agents at clinical fields^29^. There are also possibilities for new contrast mechanisms using low-field MRI. For instance, a novel susceptibility-based positive contrast technique that operates using the unique nonlinear magnetization of SPIONs at 6.5 mT has been reported^30^. Also, low-field nuclear magnetic resonance curve (NMRD) relaxometry has been used to differentiate between intracellular and extracellular distribution of ferumoxytol in tumor associated macrophages^31^.

Here, we report on the characterization of SPIONs as T_1_ contrast agents using a commercially available and FDA-approved MRI scanner at a field strength of 64 mT. We measure the properties of SPION-based agents, such as the FDA-approved ferumoxytol. We also investigate monodispersed, carboxylic acid-coated SPIONs in order to understand the size-dependent properties of iron oxides and correlate those with structural and magnetic features. We find that SPION-based contrast agents exhibit favorable qualities as T_1_ contrast agents at low-field and outperform the longitudinal relaxivity of a commercially available Gd-based agent, gadobenate dimeglumine, by nearly 9 × at room temperature and approximately 8 × at physiological temperatures. We find that longitudinal relaxivities of SPIONs at 64 mT are nearly an order of magnitude larger than at a clinical field strength of 3 T, measuring up to 67 L mmol^−1^ s^−1^. Moreover, at 64 mT, the ratio of the transverse relaxivity to the longitudinal relaxivity is of order one, indicating that transverse relaxation does not dominate SPION induced relaxation. Together with the earlier works mentioned above^9,27–31^, this work suggests that SPION-based contrast agents could play a crucial role in contrast-enhanced MRI at low-fields.

## Results

### Structural and magnetic properties of contrast agents

**Figure 1:**
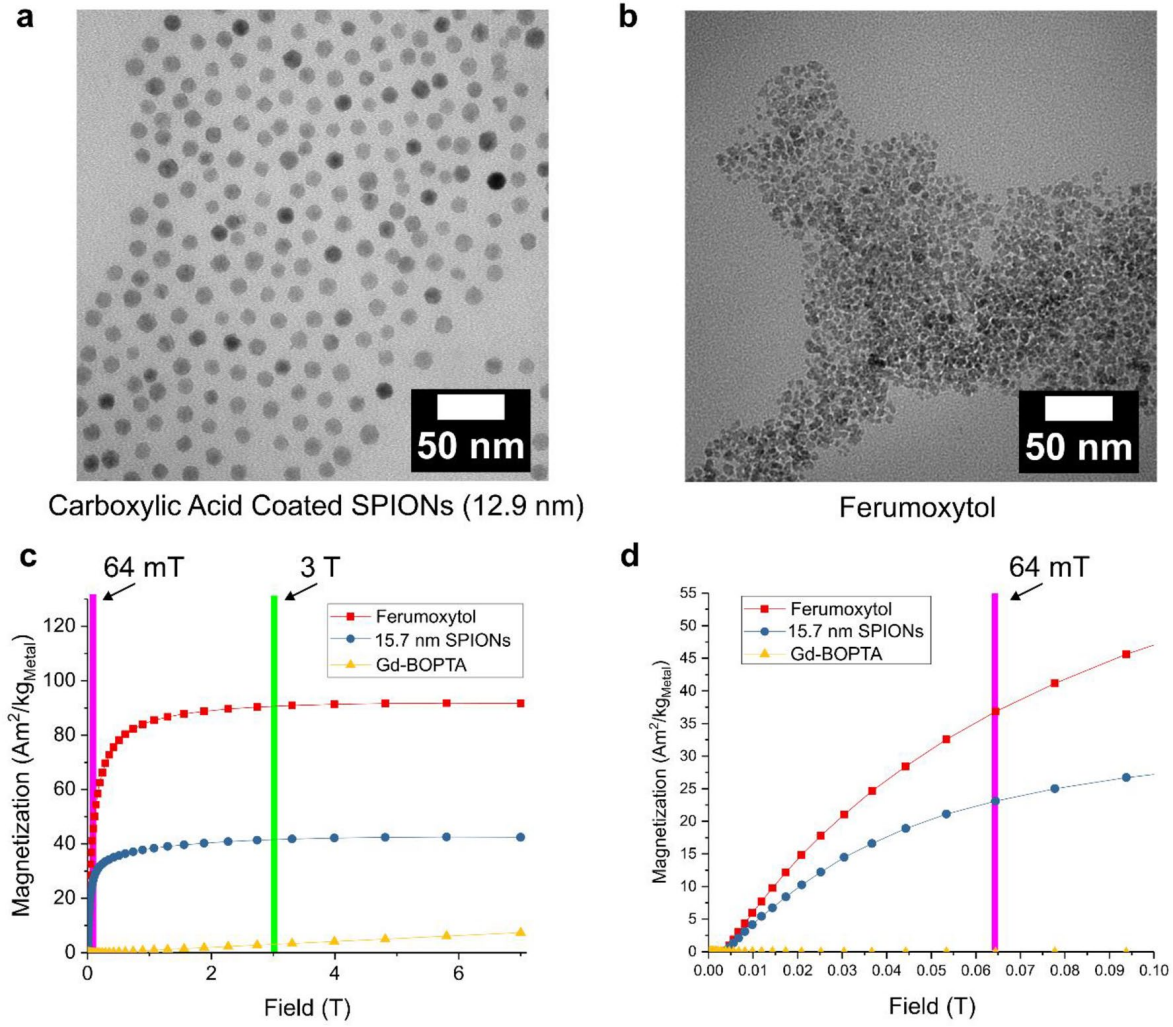
(**a**) Transmission electron microscope (TEM) image of 12.9 nm of carboxylic coated SPIONs. (**b**) TEM image of ferumoxytol. (**c**) Magnetization as a function of applied field for ferumoxytol, 15.7 nm Fe_3_O_4_ SPIONS, and Gd-BOPTA with vertical bars to show field regions corresponding to 64 mT (low-field MRI) and 3 T (standard clinical field). (**d**) Expanded region of magnetization versus applied field showing low-field regime.

Ferrimagnetic SPIONs with different sizes and coatings were used to explore T_1_ contrast for 64 mT MRI. The efficacy of contrast agents is directly related to their physical and magnetic properties. Thus, we characterized the properties of each contrast agent with electron microscopy and superconducting quantum interference device (SQUID) magnetometry to better understand their potential performance as T_1_ contrast agents. Figure 1a,b show transmission electron microscopy (TEM) images that represent the two general types of iron oxide particles used in this study. The first type are spherical, monodispersed SPIONs stabilized by a coating of carboxylic acid. Figure 1a shows an example of these particles. We acquired these types of particles (commercial vendors listed in Methods) with 4 sizes (4.9 nm, 8.5 nm, 12.9 nm and 15.7 nm) in order to investigate size-dependent contrast properties of the SPIONs. These particles have isolated iron oxide cores that are well-separated due to a combination of steric and electrostatic forces in aqueous solution. The second general class of SPIONs used in the study are therapeutic agents containing clusters of iron oxide cores with polydisperse diameter embedded in a polymer network. An example of this type of contrast agent, Feraheme (ferumoxytol), is shown in Fig. 1b. Ferumoxytol is an FDA-approved and commercially available pharmaceutical agent used for treatment of iron deficiency disorders. It is comprised of iron oxide cores embedded within a carbohydrate coat^32,33^. We also investigated a discontinued SPION-based imaging agent, Feridex (ferumoxides), which is made of an iron oxide-dextran complex^20,34^. In addition to SPIONs, we also procured an FDA-approved, Gd-based MRI contrast agent, Multihance (gadobenate dimeglumine), in order to compare the SPIONS to a commercially available Gd-chelate. Gadobenate dimeglumine (Gd-BOPTA) is a Gd-based chelate complex used for contrast-enhanced imaging of the central nervous system and magnetic resonance angiography^17^. Gd-BOPTA exhibits longitudinal relativities similar to other commercially available Gd-based contrast agents at clinical field strengths (1.5–3 T)^35^.

Figure 1c,d show room-temperature (21.5 °C) measurements of the magnetization as a function of applied magnetic field for two of the iron oxide-based agents, 15.7 nm carboxylic acid-coated nanoparticles and ferumoxytol. Figure 1d shows a magnified region of the same data in 1c from 0 to 0.1 T. The figures also show the magnetization of Gd-BOPTA for comparison. Both types of SPIONs have nonlinear magnetization curves that are characteristic of superparamagnetic particles. At zero applied field, the SPIONs show no remnant magnetization. As the applied field increases, their magnetization rapidly increases and then saturates above 2 T. At high fields, ferumoxytol shows a saturation magnetization that is comparable to bulk magnetite at room temperature (bulk Fe_3_O_4_ has *M*_*S*_ = 92 Am^2^/kg)^36^. The 15.7 nm particles show a saturation magnetization that is 46% of bulk magnetite. This reduction in magnetization is often observed in magnetite nanoparticles and is caused by crystalline defects within the particles^37,38^. The other carboxylic acid-coated particles also have a reduced saturation magnetization compared to bulk magnetite, ranging from 40.3 to 74.6 Am^2^/kg. Gd-BOPTA shows paramagnetic behavior, as expected for a Gd-chelate, and exhibits a linear increase in magnetization as a function of applied magnetic field. The magnetization of SPIONs is much higher than Gd-based agents across the measured field range (0–7 T). This is also true for the low-field regime, where the SPIONs have already reached 40% to 50% of saturation magnetization at 64 mT (Fig. 1d). Magnetization data was collected for each of the contrast agents at 21.5 °C, approximately the same temperature used for MRI experiments. For the SPIONs, each magnetization curve was fit to a single Langevin function and used to extract the effective magnetic diameter of the particles (details in Supplemental Information). Table 1 has a summary of the physical and magnetic properties of the SPIONs.

**Table 1:** Magnetic and structural properties.

Sample	Physical core diameter (nm)	Saturation magnetization (Am^2^/kg)
A	4.9 ± 0.7	40.3 ± 0.9
B	8.5 ± 0.9	74.6 ± 2.6
C	12.9 ± 1.1	44.8 ± 1.4
D	15.7 ± 1.5	42.4 ± 1.3
Ferumoxytol	–	95.7 ± 2.8
Ferumoxides	–	52.7 ± 1.1

ImageJ was used to determine core diameter from TEM^50^. The physical core diameter represents the average diameter of particles assuming a spherical core and the error is the standard deviation. Ferumoxytol and ferumoxides were excluded from the image analysis because the overlap of particles made it difficult to distinguish individual particle cores. Saturation magnetization was determined from a combination of magnetometry and ICP-OES. The error in saturation magnetization has been propagated in quadrature using experimental error from magnetometry and ICP-OES (see supplemental information for more details).

### Magnetic resonance imaging at 64 mT and 3 T

**Figure 2:**
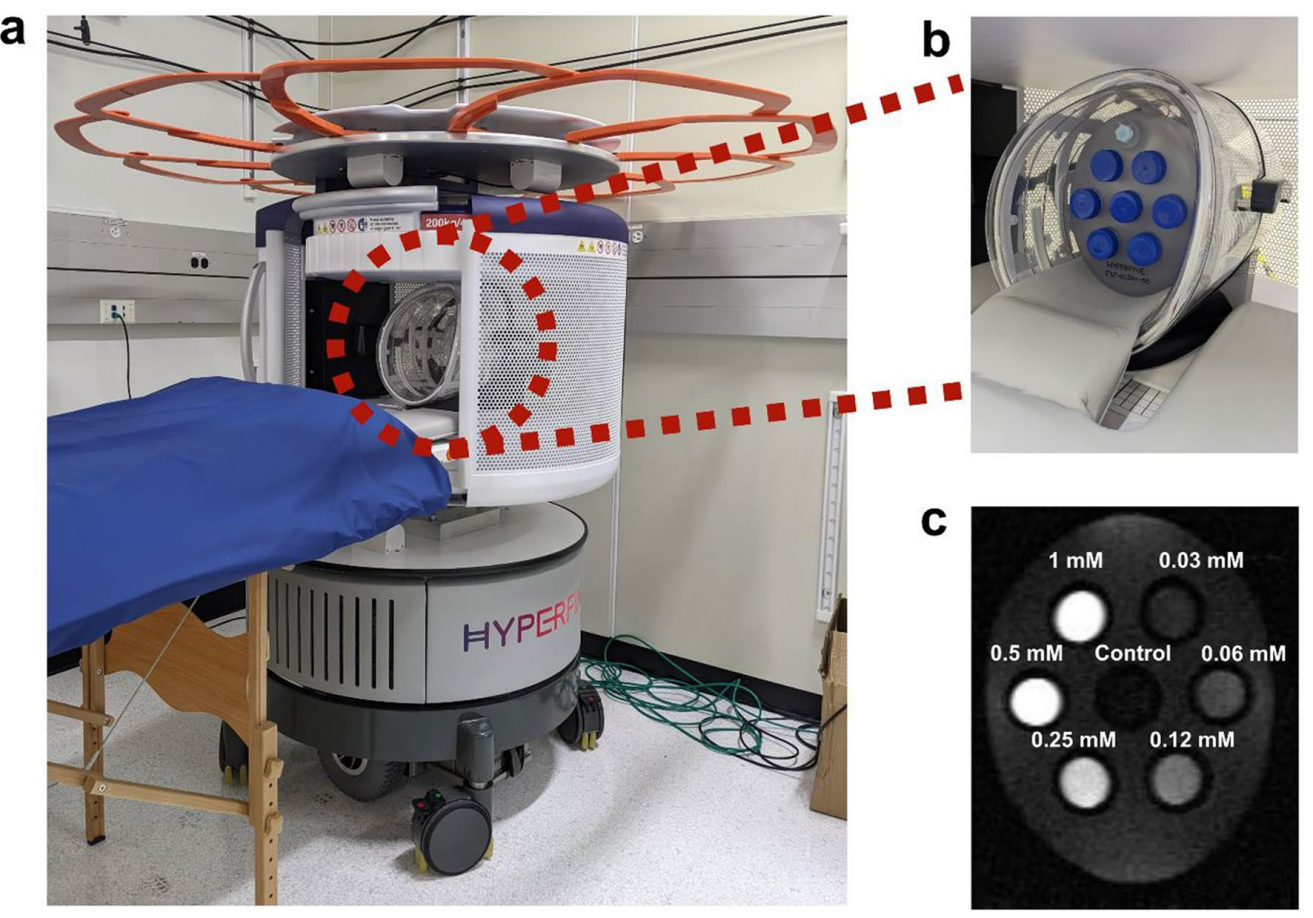
The 64 mT MRI scanner (Hyperfine, Guilford, CT) with retractable 0.5 mT field line extended above the unit. (**b**) A close-up image showing an array of samples arranged within a water-filled phantom and situated within the head coil for imaging. (**c**) A T_1_-weighted fast spin echo image showing a cross section of the phantom, containing a series of 8.5 nm SPION samples (TI = 900 ms, TE = 5.96 ms).

Contrast agents were characterized with MRI at 64 mT and 3 T to compare performance between low-field and clinical field regimes. Measurements on contrast agents were performed using MRI phantoms for imaging procedures. The 64 mT MRI scanner used for low-field imaging is shown in Fig. 2a. The image shows the scanner with the retractable 0.5 mT critical boundary (FDA requirement to meet pacemaker safety at 0.5 mT) extended out above the permanent magnet assembly and head coil. Figure 2b shows a close-up image of the head coil with a phantom for imaging an array of samples. Samples were prepared in 50 mL centrifuge tubes using dilutions of contrast agents in agarose gel and arranged in the phantom, which was custom 3D printed by Hyperfine (Guilford, CT, USA) and filled with water. Figure 2c shows a typical T_1_-weighted fast spin echo (FSE) MR image of an axial cross-section of the phantom. In this image, the phantom is filled with an array of 8.5 nm SPION solutions containing different nominal concentrations of Fe (“nominal” refers to the Fe concentration specified by the manufacturer’s label).

**Figure 3:**
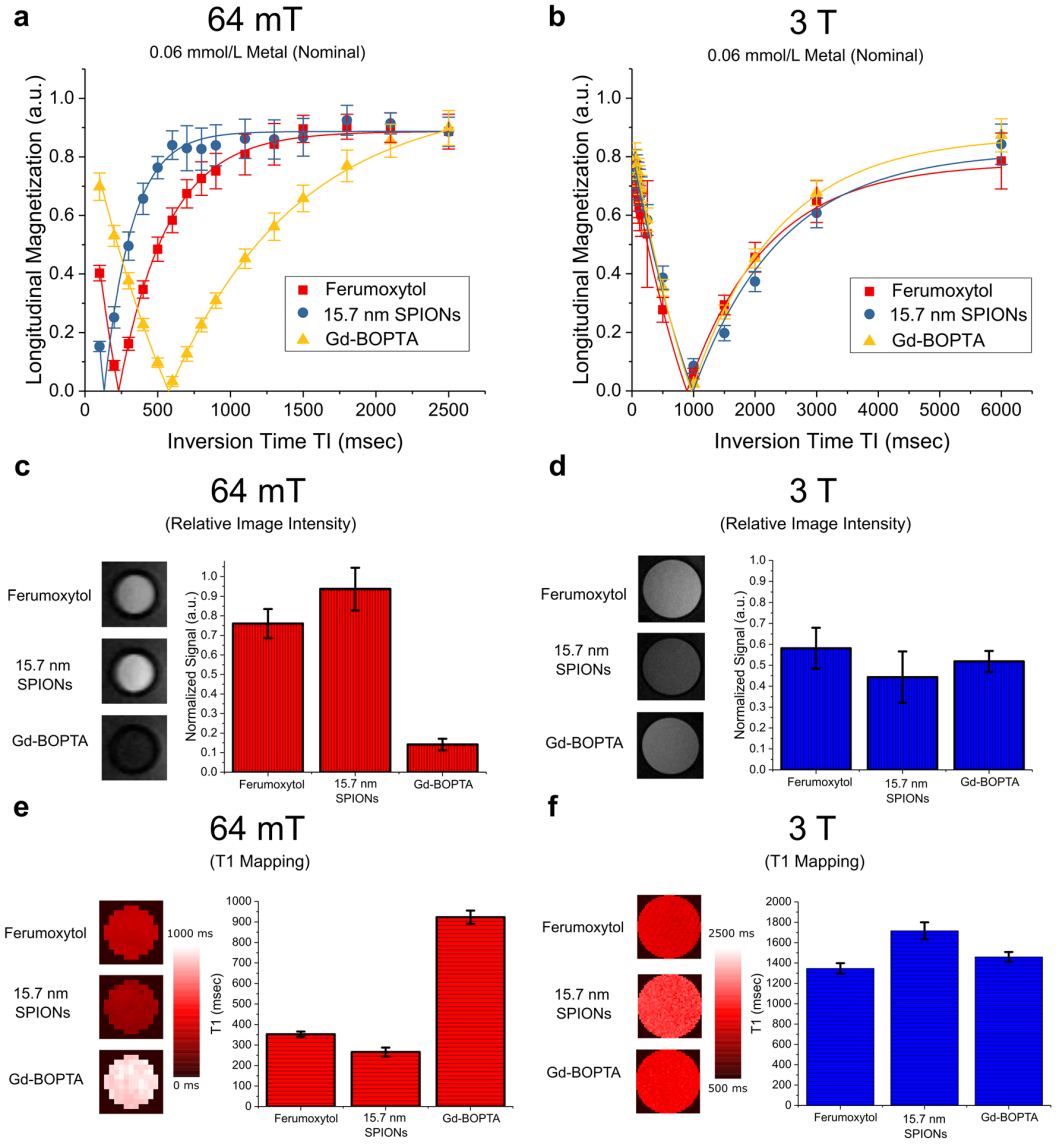
Inversion recovery curves for ferumoxytol, 16 nm SPIONS and Gd-BOPTA at (**a**) 64 mT and (**b**) 3 T. (**c**) Normalized image intensity from the 64 mT MR image corresponding to TI = 700 ms, error bars represent standard deviation of normalized intensity. (**d**) Normalized image intensity from the 3 T MR image corresponding to TI = 2000 ms, error bars represent standard deviation of normalized intensity. (**e**) T_1_ maps for corresponding ROI’s (regions of interest) at 64 mT and (**f**) T_1_ maps at 3 T.

Figure 3 shows MRI data at 64 mT and 3 T for three contrast agents—15.7 nm carboxylic acid-coated nanoparticles, ferumoxytol and Gd-BOPTA. A comparison of T_1_-weighted MRI scans at 64 mT show a pronounced difference in relaxation properties of iron oxide-based contrast agents and Gd-BOPTA. Figure 3a shows longitudinal relaxation curves using signal intensity extracted from a 64 mT inversion recovery FSE sequence (details in Methods). Each sample contained 0.06 mmol/L nominal concentration of either iron or gadolinium metal. The signal at each inversion time represents the average signal value within a region of interest (ROI) containing a cross-section of the specific sample. Error bars correspond to the standard deviation of the signal in the ROI. Solid lines were generated by fitting the data to a general inversion recovery equation (Supplemental Information). At 64 mT, the 15.7 nm SPIONs are most efficient at reducing T_1_, followed by the ferumoxytol. The Gd-BOPTA has the slowest rate of T_1_ relaxation of the three contrast agents. The significant reduction in T_1_ for the iron-oxide based agents compared to Gd-BOPTA suggests that the SPIONs are more efficient at relaxing longitudinal magnetization at 64 mT. Figure 3b shows a corresponding curve at 3 T, which was also collected using a spin echo inversion recovery sequence (details in Methods). The 3 T data is markedly different from 64 mT, showing little difference between each of the contrast agents. The difference between longitudinal relaxation observed at low fields, therefore, is not observed at higher fields commonly used for clinical imaging.

The difference between contrast agents can be further visualized by inspecting individual spin echo images used to form the inversion recovery relaxation curves. Figure 3c shows 64 mT spin echo images (inversion time, TI = 700 ms) for each contrast agent and corresponding plots of the normalized image intensity. At 64 mT, the ferumoxytol and 15.7 nm particles have signal intensities that are roughly 5 × and 6 × larger than the Gd-BOPTA, because they relax longitudinal magnetization much more efficiently. Consequently, the 15.7 nm SPIONs and ferumoxytol appear bright relative to Gd-BOPTA. Figure 3d shows MR images (TI = 2000 ms) taken at 3 T, along with plots of the normalized intensity images. The 3 T data set shows little variation in contrast as a function of added agent.

T_1_ maps at 64 mT and 3 T offer another means for quantitative comparison between the contrast agents (Fig. 3e,f). For a nominal metal concentration of 0.06 mmol/L, the 15.7 nm SPIONs and ferumoxytol have T_1_ values of 266 ms and 353 ms, respectively, at 64 mT. These values are 29% and 38% of the value for Gd-BOPTA at 64 mT, which is 923 ms. At 3 T, however, the difference between T_1_ values among contrast agents is less pronounced. These values are 1347 ms for ferumoxytol, 1716 ms for the 15.7 nm SPIONs, and 1460 ms for Gd-BOPTA.

### Relaxivity measurements at 64 mT and 3 T

To further quantify the difference between contrast agents, we measured the longitudinal and transverse relaxivities at 64 mT and 3 T. The relaxivity (*r*_*1*_ for longitudinal and *r*_*2*_ for transverse) is given by

1 $$\frac{1}{{T_{1,2} }} = \frac{1}{{T_{1,2}^{\prime } }} + r_{1,2} \cdot \left[ {CM} \right],$$

where [*CM*] is the concentration of metal (either Fe or Gd), *T*_*1,2*_ is the characteristic relaxation time of a solution containing [*CM*] and *T*′_*1,2*_ is the relaxation time for a solution without contrast agent^35^. The relaxivity was calculated by measuring the relaxation times T_1_ and T_2_ for a series of samples containing different concentrations of metal (Fe for SPIONs and Gd for Gd-BOPTA). Then, the data was fit using the linear relationship described in Eq. (1) to extract the relaxivity, which is the slope of *[CM]* versus (1/*T*_*1,2*_).

**Figure 4:**
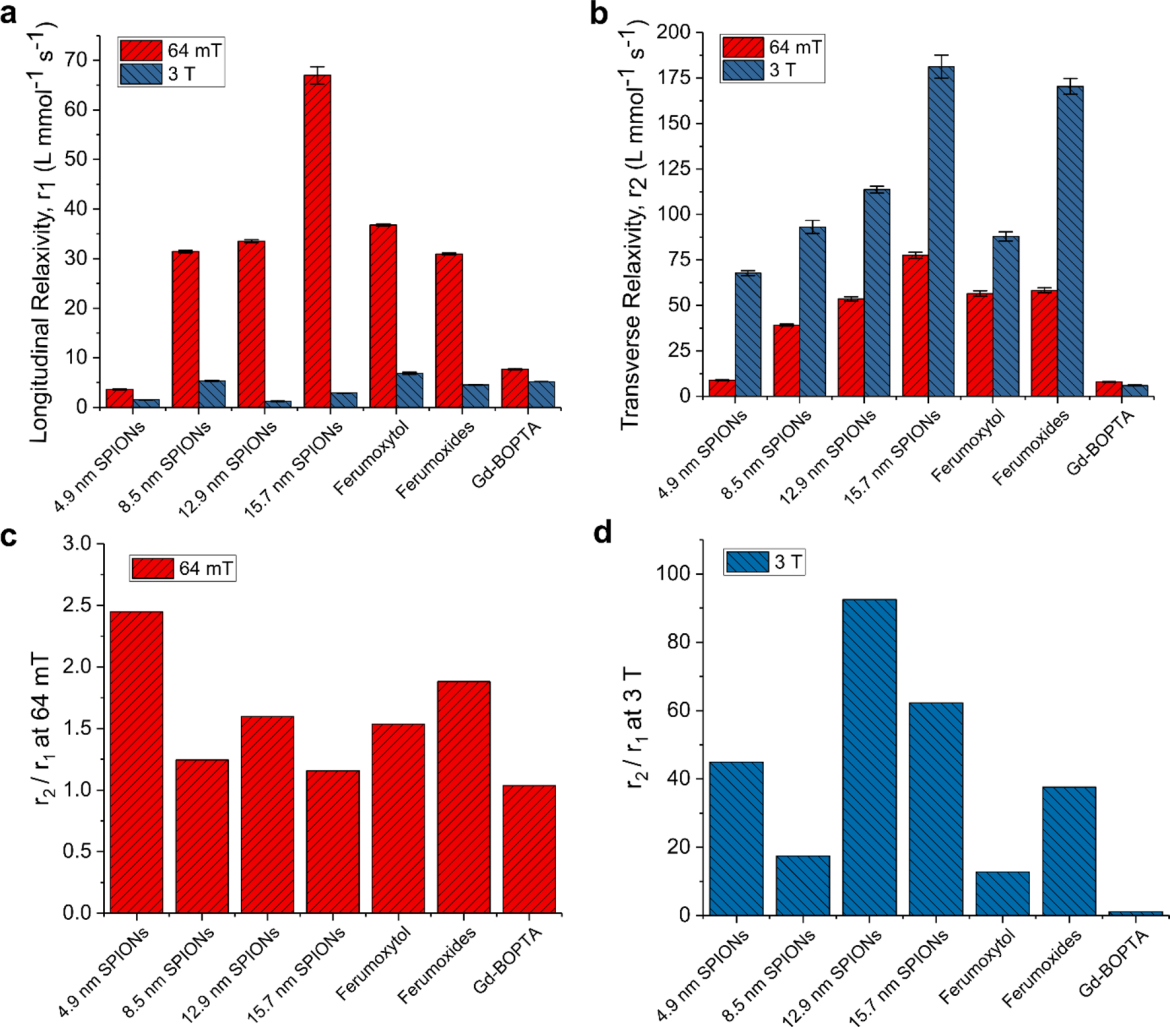
(**a**) Longitudinal relaxivity, *r*_*1*_, for each contrast agent at 64 mT and 3 T. (**b**) Transverse relaxivities, *r*_*2*_, at 64 mT and 3 T. (**c**) Ratio *r*_2_/*r*_1_ for each contrast agent at 64 mT (**d**) Ratio *r*_*2*_/*r*_*1*_ for each contrast agent at 3 T. All measurements made at 21.5 °C.

The longitudinal relaxivity, transverse relaxivity, and the ratio of r_2_/r_1_ for all contrast agents at both field strengths, 64 mT and 3 T, are plotted in Fig. 4. The data are also summarized in Tables 2 and 3. For the carboxylic acid-coated SPIONs, the longitudinal and transverse relaxivities scale approximately with their physical core sizes. So, larger particles tend to have higher relaxivities. At 64 mT, iron oxide-based contrast agents exhibit much higher r_1_ values than the Gd-BOPTA. The 15.7 nm SPIONs have the highest r_1_ at 67 L mmol^−1^ s^−1^, which is a factor of 8.7 × higher than the paramagnetic Gd-BOPTA. Ferumoxytol has an r_1_ of 36.8, which is a 4.8 × increase compared to Gd-BOPTA. In general, the longitudinal relaxivities of iron oxide-based agents at 64 mT are an order of magnitude higher than those measured at 3 T, which range from 1.2 to 6.9 L mmol^−1^ s^−1^. Therefore, the iron oxide-based contrast agents are very effective at reducing T_1_ times at 64 mT. However, the property of high r_1_ does not, by itself, mean that a contrast agent will be an effective T_1_ agent. The contrast agent must also have a transverse relaxivity that is on the same order as its longitudinal relaxivity, so that transverse relaxivity does not dominate the net relaxation. At 64 mT, the SPIONs show a considerable reduction in transverse relaxivity compared to the values at 3 T. We used the longitudinal and transverse relaxivities to calculate the ratio r_2_/r_1_ for each contrast agent. This ratio is an important indicator of efficiency as a T_1_ agent and a lower value of r_2_/r_1_ means that the T_1_ reduction effect of the contrast agent will not be dominated by T_2_/T_2_^*^ decay of signal^35^. The 64 mT ratios for all of the SPION-based contrast agents range from 1.2 to 2.4, which compares well to the value of Gd-BOPTA at 1.03 (Fig. 4c). The r_2_/r_1_ ratios for SPIONs are dramatically smaller than those measured at 3 T, which are an order of magnitude higher (Fig. 4d). A material is a good candidate for a T_1_ contrast agent if the ratio r_2_/r_1_ can be minimized while maintaining a high r_1._ At 64 mT, the SPIONs exhibit large values of r_1_ and low values of r_2_/r_1,_ suggesting that they perform very well as T_1_ agents at 64 mT. Moreover, they exhibit r_1_ values that can exceed the Gd-BOPTA by up to 8.7 × while simultaneously showing a low r_2_/r_1_. This indicates that they may be a very useful alternative to Gd-chelates in contrast-enhanced MR procedures where positive contrast is preferred.

**Table 2:** Relaxivity properties, 64 mT MRI.

Sample	r_1_, MRI (L mmol^−1^ s^−1^) 21.5 °C	r_2_, MRI (L mmol^−1^ s^−1^) 21.5 °C	r_2_/r_1_, MRI (L mmol^−1^ s^−1^) 21.5 °C	r_1_, NMRD (L mmol^−1^ s^−1^) 21.5 °C	r_1_, NMRD (L mmol^−1^ s^−1^) 37 °C
A	3.6	8.9	2.4	4.3	4.0
B	31.4	39.2	1.2	28.5	20.7
C	33.5	53.6	1.6	36.7	26.8
D	67.0	77.5	1.2	61.5	46.3
Ferumoxytol	36.8	56.5	1.5	42.2	31.5
Ferumoxides	30.9	58.3	1.9	26.0	20.4
Gd-BOPTA	7.7	7.9	1.03	6.9	5.6

**Table 3:** Relaxivity properties, 3 T MRI.

Sample	r_1_, MRI (L mmol^−1^ s^−1^) 21.5 °C	r_2_, MRI (L mmol^−1^ s^−1^) 21.5 °C	r_2_/r_1_, MRI (L mmol^−1^ s^−1^) 21.5 °C
A	1.5	67.7	44.9
B	5.3	93.1	17.4
C	1.2	113.7	92.5
D	2.9	181.3	62.3
Ferumoxytol	6.9	87.9	12.8
Ferumoxides	4.5	170.4	37.6
Gd-BOPTA	5.2	5.9	1.1

### NMRD Measurements

**Figure 5:**
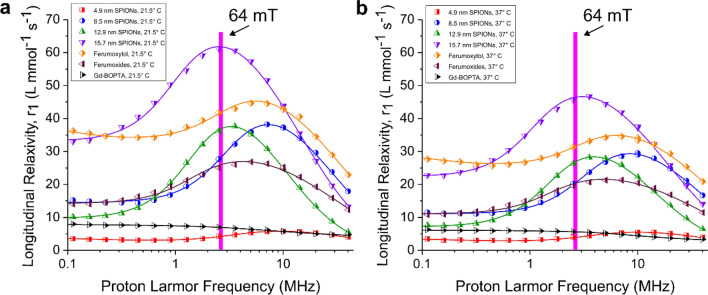
Relaxivity profiles obtained from the nuclear magnetic resonance dispersion (NMRD) data recorded at (**a**) 21.5 °C and (**b**) 37 °C. Solid lines show fits to theory for proton relaxation. The large vertical lines at 2.72 MHz correspond to the Larmor frequency of protons at 64 mT.

To further understand field dependent relaxivity of the contrast agents, we measured the nuclear magnetic resonance dispersion (NMRD) curves of each contrast agent using fast-field cycling relaxometry. The relaxivity profiles for the contrast agents are shown in Fig. 5 at 21.5 °C and 37 °C. The solids lines for the SPIONs show fits to theory describing proton relaxation in the presence of superparamagnetic nanoparticles^39,40^. These fits capture the qualitative features of the data and have the shape typically observed for solvent molecules in the presence of SPIONs. The fit for the Gd-BOPTA was performed using Solomon-Bloembergen-Morgan theory, which describes proton relaxation in the presence of paramagnetic ions^41–43^. The relaxivity peaks present from 2 to 10 MHz originate from the rise in the thermal average of the electron magnetic moment of the SPIONs with increasing magnetic field followed by a decrease at frequencies on the order of the inverse of the correlation time for translational diffusion^44,45^. Translational diffusion is the dynamic process modulating the dipole–dipole interaction between the average magnetic moment of the SPIONs and the magnetic moment of water protons, and is the dominant T_1_ relaxation process at these field values. Its correlation time (${\tau }_{D}=\frac{{d}^{2}}{D}$) depends on the distance of closest approach (*d*) between electron and proton spins and it is thus related to the SPION core size, and on the diffusional coefficient (*D*). At low magnetic fields, a further contribution to water proton relaxation is present due to the dipole–dipole interaction between the (non-averaged) electron magnetic moment of the SPIONs and the magnetic moment of water protons. This interaction is typically modulated by the Néel correlation time (${\tau }_{e}$), which is the characteristic time associated with flipping the magnetization direction of the SPION’s net magnetic moment between easy axis directions. Figure 5 shows that the frequencies of the peak maxima decrease as the diameter of the nanoparticles is increased, in agreement with the diffusion-driven mode of relaxation described above.

The relaxivity profiles show that the SPIONs exhibit enhancement of r_1_ across a range of Larmor frequencies ranging from 2 up to 20 MHz. The largest particles, 15.7 nm in diameter, have a peak in the NMRD curve that is nearly coincident with the proton Larmor frequency at 64 mT. It should be noted, though, that the proton Larmor frequency (which is proportional to the magnetic field, being equal to *γ*_*I*_*B*_0_, where *γ*_*I*_ is the proton gyromagnetic ratio) is plotted on a log scale and so the peaks are rather broad and encompass a wide range of frequencies. Values for longitudinal relaxivity corresponding to 64 mT for each curve were extracted by interpolating from the theoretical fit. These are displayed in Table 2. The longitudinal relaxivities measured at 21.5 °C generally agree with the values measured using 64 mT MRI. The values measured at 37 °C (physiological temperature) show a reduction in relaxivity compared to 21.5 °C. Still, the SPIONs have higher relaxivities at this temperature compared to Gd-BOPTA. At 37 °C, the 15.7 nm SPIONs show an 8.3 × improvement and ferumoxytol shows a 5.6 × enhancement compared to the Gd-BOPTA.

## Discussion

The relaxation properties of the SPIONs measured in this study have favorable properties for applications as T_1_ contrast agents for low-field MRI. They exhibit high r_1_ values, which can be nearly an order of magnitude larger than Gd-BOPTA for the largest particles measured here (15.7 nm). The SPIONs also have r_2_/r_1_ values that are of order one at 64 mT, meaning that effects of transverse relaxation will not dominate the net relaxation. Magnetometry shows that the SPIONs have a much larger magnetization than Gd-BOPTA at 64 mT, which contributes to their enhanced relaxivities compared to the Gd-chelate. The size range of SPIONs used in this study were chosen to represent a typical range of commercially available particles (diameters listed on label were 5 nm, 10 nm, 15 nm, and 20 nm; actual diameters were measured to be 4.9 nm, 8.5 nm, 12.9 nm, and 15.7 nm). It may be possible to achieve higher r_1_ values than those measured here by further increasing the size of particles past 15.7 nm or engineering the particles to have a higher magnetization. The 15.7 nm particles have a saturation magnetization of 42.4 Am^2^/kg, which is notably smaller than the bulk magnetite and can be attributed to defects and mixed phases of iron oxide^37,38^. Further optimization of longitudinal relaxivity may be achieved by synthesizing particles in the presence of oxygen to remove defects^38^ or by custom synthesis of larger particles.

The NMRD data suggests that SPION-based agents may also prove useful as T_1_ agents across the low-field regime, besides 64 mT. The peaks in the relaxivity profiles encompass ^1^H Larmor frequencies ranging from 1 to 30 MHz, which corresponds to field strengths of 23–705 mT. SPION-based agents may be tuned based on their size and magnetization so that they suit particular field strengths. At higher field strengths, though, the transverse relaxivities are also expected to increase, which will also increase the ratio of r_2/_r_1_. So, the design of an effective SPION-based agent for a particular field should account for the balance between effects related to both longitudinal and transverse relaxivity. The engineering of agents for particular fields can be guided by theory for proton relaxation from SPIONs. The SPIONs explored here all have NMRD curves that could be qualitatively modeled by pre-existing theories^39,40^, which were used to generate the fits to relaxivity profiles in Fig. 5. This suggests that there is a framework for understanding essential physics associated with T_1_ contrast at low fields and enables a pathway for design of contrast agents with high r_1_ and low r_2_/r_1_.

While synthesis of novel contrast agents has exciting promise, the pathway for clinical application of new contrast agents is arduous. However, the results reported here on the FDA-approved ferumoxytol have immediate clinical relevance. Ferumoxytol is currently FDA-approved for the treatment of iron deficiency but has been used off-label for clinical research on SPION-based MRI contrast. Arnold et al. have reported preliminary in vivo results at 64 mT showing contrast-enhanced cerebral vasculature in patients who received ferumoxytol injections as treatment for iron deficiency anemia^9,27^. These results suggest that ferumoxytol may be useful as a blood pool agent for low-field cerebral angiography. The measurements described in this manuscript show that ferumoxytol has a longitudinal relaxivity that is 5 × larger than Gd-BOPTA as measured by both 64 mT MRI and NMRD, suggesting that it has potential to be more sensitive than Gd-chelates. Since both ferumoxytol and the 64 mT MRI scanner are FDA-approved, there are immediate pathways available for exploring clinical applications of ferumoxytol as a low-field contrast agent. Further in vivo studies will be necessary, though, to determine whether or not the enhanced relaxivity of ferumoxytol compared to Gd-BOPTA confers sufficient advantage to be used as a T_1_ agent. Gd-chelates and ferumoxytol have very different pharmacokinetic properties, so ferumoxytol may find applications in imaging routines where Gd agents have unfavorable characteristics. For instance, ferumoxytol could be favorable in situations where blood pool agents, rather than Gd-based extracellular agents, are useful^46^. SPION-based agents also have some appeal from a toxicological standpoint since iron oxide particles are biocompatible and potentially less toxic than gadolinium^32^.

Since the measurements in this study were performed on phantoms, there are some limitations to translating the relaxivities reported here to hypothetical performance in vivo. For instance, the samples used in the study were embedded in agarose since it conferred long term stability, ensuring that identical samples could be compared between different MRI scanners. While agarose provides a hydrated, tissue-mimicking environment, it is not nearly as complex as in vivo conditions. In clinical applications, contrast agents are introduced into biological environments where a variety of pharmacokinetic factors, such as aggregation or clearance time, can have an effect on relaxivity. The relaxivity of contrast agents will also change depending on the surrounding fluid environment. It is well known, for instance, that relaxivity of contrast agents varies depending on whether they are immersed in blood, water, or plasma^35^. In order to understand how environmental changes may affect r_1_, we calculated theoretical relaxivity curves for isolated ferumoxytol particles in media with diffusion coefficients corresponding to different bio-fluids and tissues, such as blood, cerebrospinal fluid, white matter, and grey matter (Supplemental Information). The calculations were performed using the same model as the fits to NMRD data. The theoretical curves show an enhancement of relaxivity in tissues with lower diffusion coefficients, meaning that r_1_ may show a diffusion-based enhancement for certain types of tissue.

Also, the 64 mT MRI measurements were performed at laboratory room temperature (21.5 °C). The performance of the contrast agents will change at physiological temperatures (37 °C). The NMRD data taken at 37 °C, however, suggest that the SPION-based contrast agents have significant longitudinal relaxivity compared to Gd-BOPTA at physiological temperature. While the phantom environments measured in this study do not simulate in vivo conditions perfectly, the measurements still provide valuable information for understanding the potential of SPION-based agents at low-field.

In addition, the toxicity profiles of SPION-based contrast agents will affect their biological uptake and will impact applications as contrast agents. Toxicity can be influenced by a variety of parameters, such as nanoparticle size or surface coating. Therefore, if high relaxivity SPIONs are developed specifically for future low-field applications, in vitro or in vivo toxicity tests will be needed to gauge the safety and efficacy of new nanoparticle formulations. Previous studies on the toxicity of SPION formulations for biological applications and can be used to guide future design efforts for low-field contrast agents^47–49^.

In conclusion, we have characterized SPION-based contrast agents with several techniques with the aim of evaluating their effectiveness as positive contrast T_1_ agents for 64 mT MRI. We focused on the field strength of 64 mT because of the emergence of an FDA approved MRI scanner at this field. Different types of SPIONs were measured using 64 mT MRI and compared to 3 T MRI. Measurements with 64 mT MRI show that SPIONs have much higher longitudinal relaxivity (9x) compared to a commercially available Gd-based agent at room temperature while also exhibiting low r_2_/r_1_ ratios. NMRD data was recorded at several temperatures, including physiological temperature, to better understand the field-dependent and temperature-dependent properties of the contrast agents. We found that at physiological temperatures, ferumoxytol, an FDA-approved SPION-based therapeutic agent, showed a 5.6 × improvement in r_1_ over Gd-BOPTA. Carboxylic-acid coated SPIONs with a diameter of 15.7 nm did even better, showing an 8.3 × enhancement. The relaxivity profiles could be fit to existing theories for proton relaxation in the presence of SPIONs. Taken together, the measurements suggest that SPIONs can play a potentially important role as positive contrast T_1_ agents for emerging applications in low field-MRI.

## Methods

### Materials

Carboxylic acid-coated iron oxide nanoparticles were purchased from Sigma Aldrich and MK Nano. Specifically, the 4.9 nm, 8.5 nm, and 15.7 nm particles were acquired from Sigma Aldrich (the labels report diameters of 5 nm, 10 nm, and 20 nm, respectively). The 12.9 nm particles were acquired from MK Nano (listed as 15 nm particles). Agarose (BioReagent for molecular biology, low electroendosmosis) was also purchased from Sigma Aldrich.

### MRI sample preparation

Samples were prepared by diluting contrast agents in an agarose gel (prepared with mass fraction of 1% agarose in H_2_O) based on the concentrations listed on the manufacturers label. Samples were prepared with concentrations of 0.03 mmol/L, 0.06 mmol/L, 0.12 mmol/L, 0.25 mmol/L, 0.5 mmol/L and 1 mmol/L of metal (Fe for the SPIONs and Gd for Gd-BOPTA).

### Transmission electron microscopy (TEM)

TEM was performed using a Tecnai T12 Spirit BT microscope with a LaB6 filament. Samples were prepared by diluting nanoparticles in a 1:1 volume ratio mixture of isopropyl alcohol and H_2_O. The diluted samples were added dropwise to the top of Formvar/carbon-coated copper grids purchased from Ted Pella. Physical core sizes of particles were analyzed using TEM images and ImageJ^50^.

### 3 Tesla MRI

An Agilent preclinical scanner was used for 3 T MRI. T_1_ measurements were made using an inversion recovery sequence with a 256 × 256 matrix and a 128 mm × 128 mm field of view. Images were acquired from 6 axial slices with a thickness of 2 mm and a gap spacing of 4 mm. The inversion times used for the sequence were 50 ms, 75 ms, 100 ms, 125 ms, 250 ms, 500 ms, 1000 ms, 1500 ms, 2000 ms, 3000 ms, and 6000 ms. The repetition time was 10000 ms and the echo time was 13.92 ms. T_2_ measurements were performed using a spin echo sequence with the same resolution, field of view, and slice parameters as the T_1_ measurements. Echo times used for T_2_ acquisition were 14 ms, 28 ms, 56 ms, 112 ms, and 224 ms. The repetition time was 10000 ms. Measurements were made using a temperature-controlled phantom. Temperature control was achieved using a closed flow loop of a perfluorocarbon coolant and monitored by a fiber optic sensor. The temperature was set to 21.5 °C in order to match the lab temperature of the 64 mT MRI measurements.

### 64 mT MRI

A Hyperfine Swoop scanner with hardware version 1.8 and software version rc8.3.1, was used to acquire T_1_ and T_2_ measurements at 64 mT. All scans used an 8-channel receive, 1-channel transmit head coil. The. T_1_ measurements were made using a research version of the Hyperfine proprietary T_1_-weighted inversion recovery 3D fast spin echo (FSE) sequence with a 220 mm × 180 mm × 180 mm field of view, an in-plane resolution of 1.6 mm × 1.6 mm, and a slice thickness of 5 mm. The inversion times used for the sequence were 100 ms, 200 ms, 300 ms, 400 ms, 500 ms, 600 ms, 700 ms, 800 ms, 900 ms, 1100 ms, 1300 ms, 1500 ms, 1800 ms, 2100 ms, and 2500 ms. The repetition time was 3000 ms and the echo time was 5.96 ms. T_2_ measurements were performed using a research version of the Hyperfine proprietary T_2_-weighted 3D FSE sequence with a 220 mm × 180 mm × 180 mm field of view, an in-plane resolution of 1.5 mm × 1.5 mm, and a slice thickness of 5 mm. Echo times used for T_2_ acquisition were 37 ms, 111 ms, 184 ms, 259 ms, 333 ms, 407 ms, 480 ms, 554 ms, 628 ms, and 702 ms. The repetition time was 3000 ms. Measurements were performed at an ambient lab temperature of 21.5 °C.

### Concentration measurements

The Fe concentration of each SPION-based contrast agent was measured using inductively coupled plasma optical emission spectroscopy (ICP-OES). ICP-OES was performed using a Perkin Elmer Optima 8300 ICP-OES optical system with a segmented-array charge-coupled device detector. Briefly, samples were digested using nitric acid, then further diluted to generate samples for measurement with ICP-OES. A detailed description of sample preparation is given in the Supplemental Information.

### T_1_/T_2_ mapping and relaxivity analysis

Samples were prepared with nominal concentrations (diluted according to concentration on manufacturer’s label) of 0 mmol/L, 0.03 mmol/L, 0.06 mmol/L, 0.12 mmol/L, 0.25 mmol/L, 0.5 mmol/L and 1 mmol/L. For measurements at 3 T, the 0 mmol/L sample was not measured because the 3 T sample holder accommodates one fewer sample than 64 mT. The concentration of the SPIONs were measured using ICP-OES and the nominal concentrations were rescaled to reflect the actual concentration of the samples (see Supplemental Information for more details). Samples were prepared by embedding contrast agents in an agarose solution (prepared with mass fraction of 1% agarose in H_2_O), which was allowed to set into a semi-solid gel at room temperature. An agarose medium was chosen for imaging because it provided long term colloidal stability, so that the same samples could be scanned at 64 mT and 3 T. Diluting the samples in other solutions did not confer the same degree of colloidal stability (see Supplemental Information). T_1_ was calculated for each voxel using LMFIT in Python for the inversion-recovery model,

2 $$S_{i} = S_{o} \left| {1 - \left( {1 + d} \right)e^{{ - {\raise0.7ex\hbox{${TI}$} \!\mathord{\left/ {\vphantom {{TI} {T_{1} }}}\right.\kern-0pt} \!\lower0.7ex\hbox{${T_{1} }$}}}} + e^{{ - {\raise0.7ex\hbox{${TR}$} \!\mathord{\left/ {\vphantom {{TR} {T_{1} }}}\right.\kern-0pt} \!\lower0.7ex\hbox{${T_{1} }$}}}} } \right|,$$

with T_1_ the target value to fit, inversion time *TI*, repetition time *TR*, scale factor for imperfect inversion *d*, the nominal signal intensity for a voxel *S*_*0*_, and measured signal intensity *S*_*i*_. At 64 mT, T_2_ maps were acquired using a research version of the Hyperfine proprietary T_2_-weighted 3D FSE sequence. The T_2_ map was calculated in the Hyperfine protocol using SciPy optimize curve_fit in Python for the model,

3 $$S_{i} = S_{o} e^{{ - {\raise0.7ex\hbox{${TE}$} \!\mathord{\left/ {\vphantom {{TE} {T_{2} }}}\right.\kern-0pt} \!\lower0.7ex\hbox{${T_{2} }$}}}} ,$$

with T_2_ the target value for the fit, echo time *TE*, nominal signal intensity without relaxation *S*_*0*_, and measured signal intensity *S*_*i*_. At 3 T, the T_2_ maps were found by using LMFIT for each voxel using Eq. (3).

After T_1_ and T_2_ were measured for all concentrations and field strengths, the data was fit using Eq. (1) to calculate the relaxivities. Unfortunately, the experimental protocol at 64 mT could not accurately measure T_1_ values less than the first inversion time of 100 ms. At high concentrations (0.25–1 L mmol^−1^ s^−1^), some of the SPION-based agents with larger physical core sizes had T_1_ values lower than 100 ms. So, we instituted a selection rule to exclude cases where the concentration of contrast agents was too high for accurate determination of measurement times. The rule states that if the T_1_ value calculated using a fit of Eq. (2) was less than the first inversion time (100 ms for 64 mT), then the data was excluded from the relaxivity fit (see Supplemental Information for more details). The data corresponding to these concentrations was then excluded from fitting the relaxivities at other conditions (r_2_ at 64 mT, r_1_ at 3 T and r_2_ at 3 T) to maintain consistency across all field strengths and relaxation types. Out of 42 measurements (6 concentrations for 7 different agents), this rule eliminated 11 data sets from relaxivity analysis and fitting. The data sets that were eliminated corresponded to samples with large nanoparticle sizes at high concentrations, since these tended to relax signal very rapidly. A table has been included in the Supplemental Information to indicate the samples that were excluded on this basis.

### SQUID magnetometry

The magnetization of samples was measured using a Quantum Design SQUID MPMS 3 magnetometer. Samples were immobilized in a water-soluble hydrogel and cured with UV light to prevent magnetic field-induced chaining and aggregation of particles during measurement.

### NMRD

Water relaxation profiles were acquired with a Stelar Spinmaster FFC2000-1 T relaxometer by measuring the water proton relaxation rates as a function of the applied magnetic field (0.01–40 MHz proton Larmor frequency). The field-cycling technique is used to detect the magnetization decay/recovery curves by acquiring the free induction decay signals from the sample after exposure to a given magnetic field for 16 different intervals of time^51^. By changing the magnetic field, longitudinal relaxation rates can be determined at all frequencies permitted by the instrument. For magnetic fields lower than 0.35 T, a 1 T pre-polarization field was applied before the measurement to generate sufficiently large signals for NMRD acquisition. The relaxation measurements, obtained from the fit of the magnetization decay/recovery curves against a mono-exponential function, were affected by an error below ± 1%. Samples were prepared with a nominal metal concentration of 1 mmol/L (using concentration specified by manufacturer) in an agarose gel (prepared with mass fraction of 1% agarose in H_2_O). The relaxivity profiles were obtained by normalization of the measured relaxation data to the metal concentration (measured by ICP-OES) after subtracting the diamagnetic relaxation rate contribution from the agarose (NMRD profiles of the agarose are included in the Supplemental Information). The measurements were performed at 21.5 °C for comparison with the MRI data and 37 °C to evaluate relaxivity at physiological temperature. Additional measurements were performed at 15 °C and 25 °C and are included in the Supplemental Information.

## Supplementary Information

Supplementary Information.

## Data Availability

The data from the study are available from the corresponding author upon reasonable request.
