# Supplementary material for: Iron oxide nanoparticles as positive T1 contrast agents for low-field magnetic resonance imaging at 64 mT
Source: Sci Rep. 2023 Jul 17;13:11520. doi: 10.1038/s41598-023-38222-6 (PMC10352268; doi:10.1038/s41598-023-38222-6)
Supplement: Supplementary file 1 — Supplementary Information. [file 41598_2023_38222_MOESM1_ESM.docx]

Supplemental Information: Iron Oxide Nanoparticles as Positive T_1_ Contrast Agents for Low-Field Magnetic Resonance Imaging at 64 mT

**Authors**

Samuel D. Oberdick^1,2*^, Kalina V. Jordanova^2^, John T. Lundstrom^1,2^, Giacomo Parigi^3^, Megan E. Poorman^4^, Gary Zabow^2^, Kathryn E. Keenan^2^

**Affiliations**

1. Department of Physics, University of Colorado, Boulder, Colorado 80309, USA
2. National Institute of Standards and Technology, Boulder, Colorado 80305, USA
3. Magnetic Resonance Center (CERM), University of Florence, via Luigi Sacconi 6, Sesto Fiorentino, 50019 Italy; Department of Chemistry “Ugo Schiff”, University of Florence, via della Lastruccia 3, Sesto Fiorentino, 50019 Italy; and Consorzio Interuniversitario Risonanze Magnetiche Metallo Proteine (CIRMMP), via Luigi Sacconi 6, Sesto Fiorentino, 50019 Italy
4. Hyperfine, Inc., Guilford, Connecticut, USA

Contents

1. Sample Key
2. Physical Core Size from Transmission Electron Microscopy (TEM)
3. Iron Concentration Measurements
4. Magnetometry
5. General Inversion Recovery Equation for Fits in Figure 3 of Main Paper
6. Relaxivity Analysis
7. Nuclear Magnetic Resonance Dispersion (NMRD) Details
8. Colloidal Stability of Contrast Agent Samples
9. Comparison of Relaxivity in Water and Agarose Gel
10. Calculation of Relaxivity Curves with Diffusion Coefficients Corresponding to Different Bio-Environments Using Relaxation Model from NMRD Analysis

**Disclaimer**: Any mention of commercial products is intended solely for fully detailing experiments; it does not imply recommendation or endorsement by NIST.

A. Sample Key

The superparamagnetic iron oxide nanoparticle (SPION) samples are referred to as Sample A, Sample B, Sample C, and Sample D throughout the supplemental information. Below is a key for identifying each sample with its corresponding physical core diameter. The commercial vendor that samples were purchased from, as well as the nominal diameter (i.e., the size listed on the label) are also given in the table.

Table S1: Sample key.

| **Sample Name** | **Physical Core Diameter (nm)** | **Nominal Diameter**  **(nm)** | **Vendor** |
| --- | --- | --- | --- |
| **A** | 4.9 ± 0.7 | 5 | Sigma Aldrich |
| **B** | 8.5 ± 0.9 | 10 | Sigma Aldrich |
| **C** | 12.9 ± 1.1 | 15 | MK Nano |
| **D** | 15.7 ± 1.5 | 20 | Sigma Aldrich |

B. Physical Core Size from Transmission Electron Microscopy (TEM)

The physical core diameter represents the average diameter of particles assuming a spherical core and the error is the standard deviation from measurements of N number of particles. ImageJ was used to determine core diameter from TEM^1^. Ferumoxytol and ferumoxides were excluded from the image analysis because the overlap of particles made it difficult to distinguish individual particle cores. The nanoparticles in Sample C (acquired from a different vendor from A, B and D) dispersed more uniformly on the carbon-coated TEM grids than samples A, B and D. This made it easier to acquire greater particles statistics for this sample, reflected in the increase in N for sample C.

Table S2: TEM Details.

| **Sample Name** | **Physical Core Diameter (nm)** | **N**  **(Particle Number)** |
| --- | --- | --- |
| **A** | 4.9 ± 0.7 | 18 |
| **B** | 8.5 ± 0.9 | 29 |
| **C** | 12.9 ± 1.1 | 2948 |
| **D** | 15.7 ± 1.5 | 40 |

C. Iron Concentration Measurements

**Sample Preparation.** The iron concentration of each sample was determined using inductively coupled plasma optical emission spectroscopy (ICP-OES). Samples were prepared by pipetting 50 μL of contrast agent into a glass scintillation vial. The solution was dried at room temperature and in laboratory atmosphere, leaving behind a precipitate of iron oxide nanomaterial. Then, 2.5 mL of nitric acid (69 % volume fraction, Rocky Mountain Reagents) were pipetted into the vial to digest the iron oxide. The samples were digested for at least 24 h. Then, the solution was diluted by transferring the 2.5 mL of digested nitric acid to a 50 mL volumetric flask. Then the volume was brought up to the 50 mL mark with deionized (DI) water. The solution was mixed at points during transfer by swirling the contents of the flask to ensure that the digestion was uniformly distributed and diluted. Finally, approximately 15 mL of the final solution were used for ICP-OES measurement.

**ICP-OES Measurements.** ICP-OES was performed using a Perkin Elmer Optima 8300 ICP-OES optical system with a segmented-array charge-coupled device detector. The ICP-OES system is calibrated using ICP Quality Control Standard #1 (Catalog #QCS-01-1, AccuStandard). The system is also tested using two quality control checks. First, a quality control test for low concentrations is performed using the 1643F NIST Standard (LGC Standards). Then, a high concentration test is performed using the 25 Component ICP CCV Standard A, which is traceable to the NIST SRM 3100 series (High-Purity Standards). For 5 of the samples (Sample A, Sample B, Sample C, Sample D, Sample E, and Feridex (ferumoxides) a single vial with approximately 15 mL of digested Fe solution was measured. The measurement gives the concentration of Fe and relative standard deviation (RSD). The RSD was taken as the uncertainty for each measurement. For Feraheme (ferumoxytol), two vials containing approximately 15 mL of solution were measured, and the concentration was found by taking an average of the two measurements. The net uncertainty was taken by adding the RSD of each measurement in quadrature and dividing by two (from the average),

|  | $\delta_{Feraheme}=\frac{1}{2}\sqrt{\left( \delta_{measuremt 1} \right)^{2}+\left( \delta_{measuremt 2} \right)^{2}}$. | (S1) |
| --- | --- | --- |

Table S3. Fe concentration for each sample and the corresponding uncertainty.

| **Sample Name** | **Iron Concentration (mg/mL)** | **Uncertainty (mg/mL)** |
| --- | --- | --- |
| Sample A | 4.307 | 0.041 |
| Sample B | 2.087 | 0.039 |
| Sample C | 3.850 | 0.050 |
| Sample D | 5.749 | 0.035 |
| Sample E | 0.697 | 0.029 |
| Ferumoxides | 10.548 | 0.067 |
| Ferumoxytol | 28.430 | 0.293 |

D. Magnetometry

**M-H Curves.** Magnetometry was performed on a Quantum Design MPMS3 magnetometer. Major loops were collected by sweeping the field from +7 T → 0 T → -7 T → 0 T → +7 T. The field was swept at a rate of 10 mT/s and data were collected with uniform spacing in log(Field). The data was collected at 294.65 K to match the acquisition temperatures for MR relaxivity data.

**Sample Preparation and Iron Oxide Content**. Nanoparticle samples were immobilized in an ultraviolet (UV) curable matrix of poly(ethylene glycol) dimethacrylate (PEGDMA) (average M_n_ 750, contains 900 ppm – 1100 ppm MEHQ as inhibitor, Sigma Aldrich). A stock solution of UV-curable PEGDMA precursor was made by mixing a volume fraction of 70 % PEGDMA 750 with a volume fraction of 30 % H_2_O. Then, a mass fraction of 1 % lithium phenyl-2,4,6-trimethylbenzoylphosphinate (LAP) (Sigma Aldrich) was dissolved in the solution. The SQUID samples were prepared by mixing NP stock solution with the following amounts of UV-curable precursor:

Table S4. Dilution volumes for magnetometry sample preparation.

| **Sample Name** | **Contrast Agent Stock Volume** | **UV-Curable Precursor Volume** |
| --- | --- | --- |
| Sample A | 10 μL | 80 μL |
| Sample B | 10 μL | 80 μL |
| Sample C | 10 μL | 55 μL |
| Sample D | 10 μL | 80 μL |
| Ferumoxides | 10 μL | 190 μL |
| Ferumoxytol | 10 μL | 527 μL |

From the samples mixed above, 80 μL was added to a small gel capsule and cured using a 365 nm UV lamp under a N_2_ environment for 3 minutes. The initial stock concentrations of each sample, as determined by ICP-OES, were used to calculate the concentration of Fe_3_O_4_ in each sample used for SQUID magnetometry. An Fe_3_O_4_ stoichiometry was assumed to convert from mass of iron to mass of iron oxide/magnetite.

Table S5. Mass of Fe_3_O_4_ and corresponding error for magnetometry samples.

| **Sample Name** | **Total Fe_3_O_4 ­_mass (g)** | **Error in Fe_3_O_4 ­_mass (g)** |
| --- | --- | --- |
| Sample A | 5.291 × 10^-5^ | 5.078 × 10^-7^ |
| Sample B | 2.563 × 10^-5^ | 4.84 × 10^-7^ |
| Sample C | 6.549 × 10^-5^ | 8.506 × 10^-7^ |
| Sample D | 7.062 × 10^-5^ | 4.253 × 10^-7^ |
| Ferumoxides | 5.831 × 10^-5^ | 3.679 × 10^-7^ |
| Ferumoxytol | 5.853 × 10^-5^ | 6.029 × 10^-7^ |

**Background Subtraction.** To separate the iron oxide magnetization from the diamagnetic contribution of the polymer matrix, we assumed that the magnetization of the polymer matrix and the magnetization of the superparamagnetic particles added linearly to form the magnetization measured by the magnetometer,

|  | $M_{sample}=M_{SPIONS}+M_{polymer}$. | (S2) |
| --- | --- | --- |

We also assumed that the magnetization of the polymer matrix was linearly proportional to the applied magnetic field,

|  | $M_{polymer}=\chi_{polymer}H,$ | (S3) |
| --- | --- | --- |

where 𝜒*_polymer_* is the magnetic susceptibility of the polymer matrix. To extract the superparamagnetic contribution of the particles, the high field data was fit to a linear background. The linear background was then subtracted away from the data, isolating the magnetization from the SPIONS. The effect of the background correction is show in Figure S1.


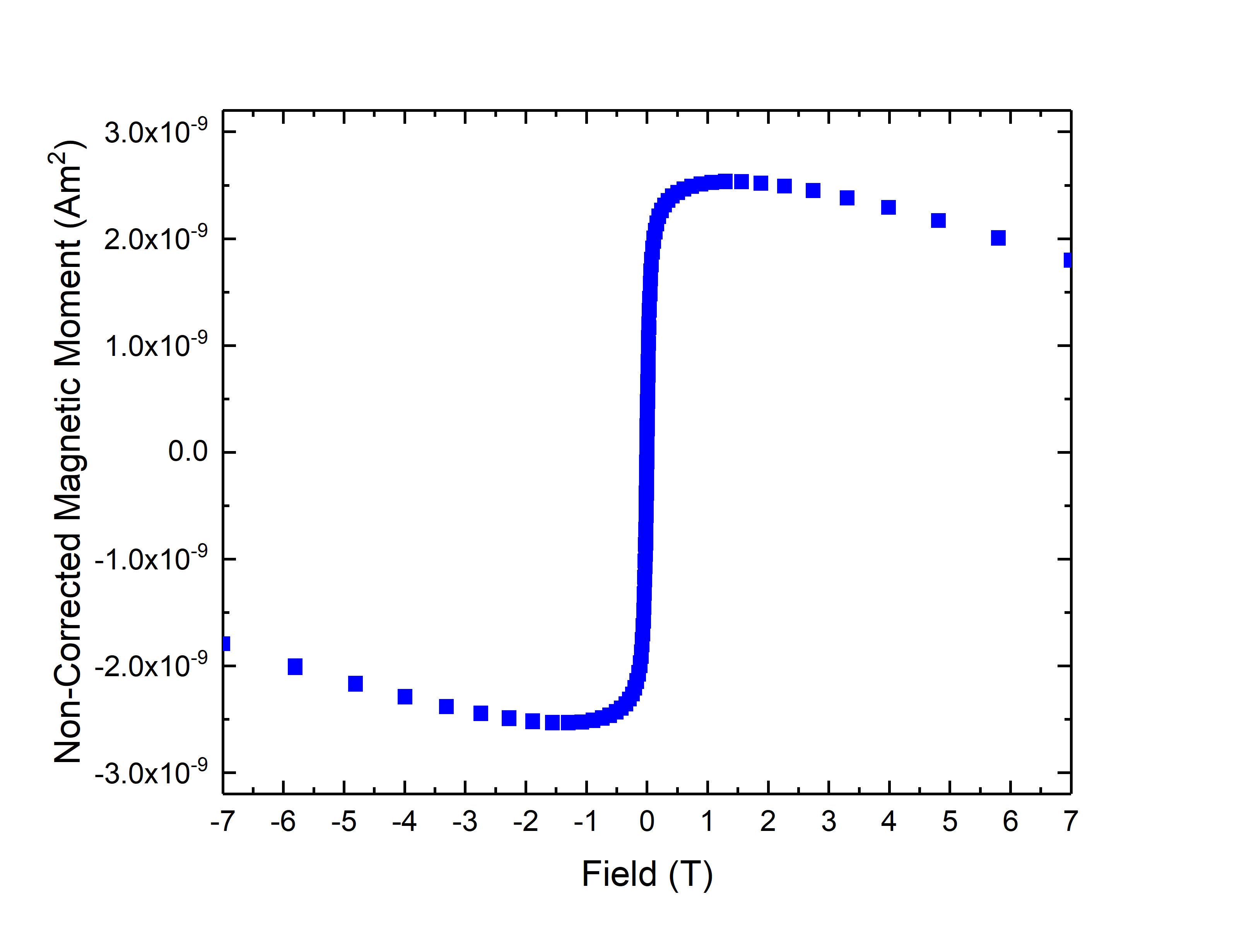

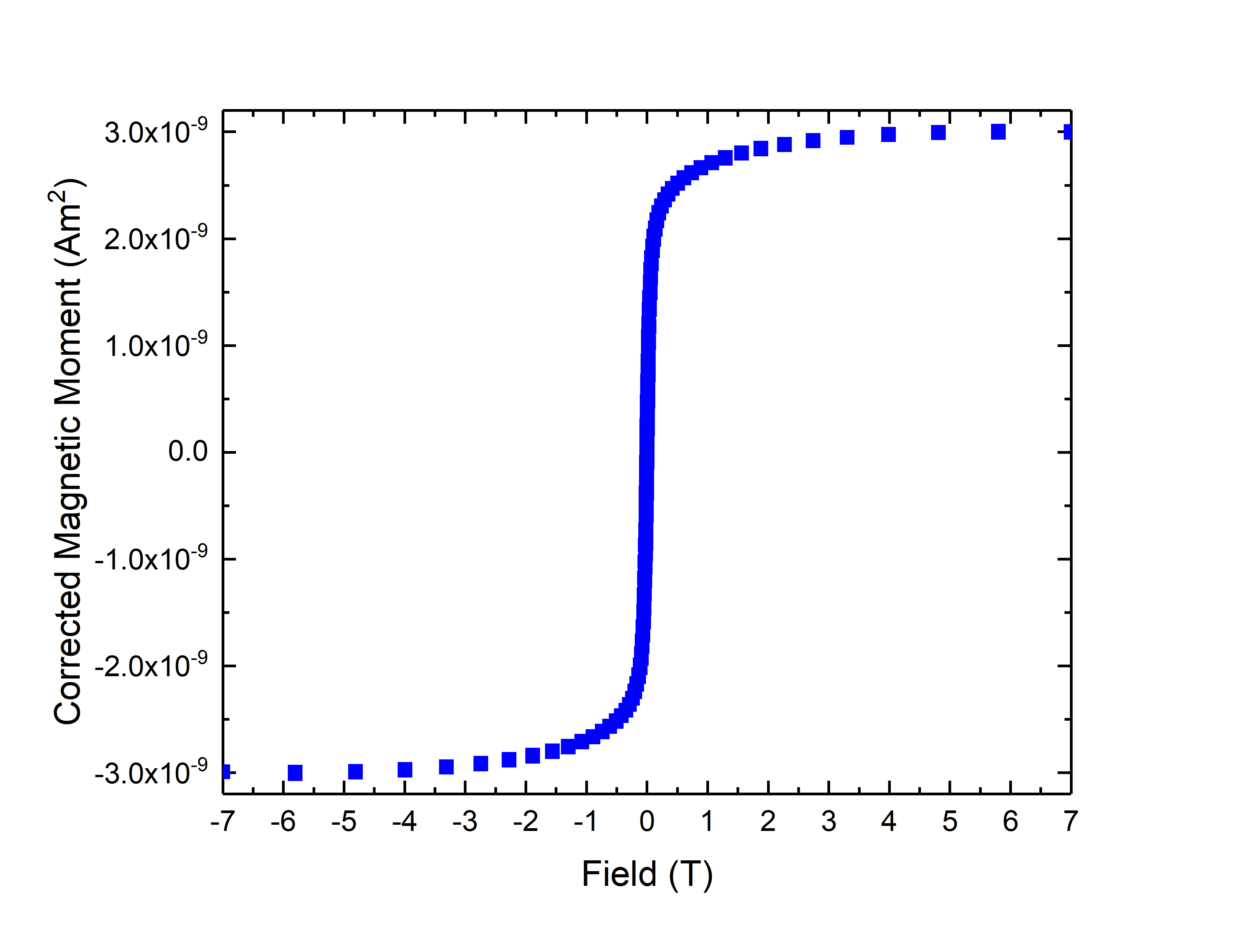


Figure S1: Example of polymer background correction on magnetometry data from 15.7 nm SPIONS. Original data are on the left and the corrected data are on the right.

**Saturation Magnetization.** After subtraction of the polymer background, the saturation magnetization of each sample was found by dividing the magnetic moment of the samples measured at 7 T by the mass of iron oxide in the sample. The mass of iron oxide was calculated using the mass of iron measured by ICP-OES and an assumption of magnetite (Fe_3_O_4_) stoichiometry. The error in the saturation magnetization was found by error propagation from the error in the moment (given by the MPMS3) and the error in the mass of the iron oxide.

**Langevin Fitting.** Following the background subtraction, the magnetization data from the SPIONS were fit to a Langevin function of the form

|  | $M_{SPIONS}\left( \alpha H \right)=M_{0}\left( \coth\left( \alpha H \right)-\frac{1}{\alpha H} \right),$ | (S4) |
| --- | --- | --- |

with the variable *α* is given as

|  | $\alpha=\frac{m}{k_{B}T},$ | (S5) |
| --- | --- | --- |

where *M_0_* is the saturation magnetization of the sample, *H* is the applied field, *m* is the magnetic moment of a single iron oxide nanoparticle, *k_B_* is Boltzmann’s constant, and *T* is the temperature. Fitting was done using a custom nonlinear curve fitting function in OriginPro2017. If we assume that the particles have a spherical shape and uniform density, then α can be expressed as

|  | $\alpha=\frac{m}{k_{B}T}=\frac{\rho M_{s}V}{k_{B}T}=\frac{{\rho M}_{s}}{k_{B}T}\cdot\frac{4}{3}\pi r^{3},$ | (S6) |
| --- | --- | --- |

where *M_s_* is the (mass) magnetization of the iron oxide nanoparticles and *V* is the volume of the particles (equal to (4/3)π*r*^3^ if spherical). Rearranging equation (S6) to express the diameter, *d,* in terms of the other parameters gives

|  | $d=2r=2\left( \frac{{3\alpha k}_{B}T}{4\pi\rho M_{s}} \right)^{1/3}$. | (S7) |
| --- | --- | --- |

Equation (S7) was evaluated for each sample to determine the magnetic diameter of the particles.

The error in the diameter of the particles was found using error propagation,

|  | $\frac{\delta d}{d}=\frac{1}{3}\left( \left( \frac{\delta\alpha}{\alpha} \right)^{2}+\left( \frac{\delta M_{s}}{M_{s}} \right)^{2} \right)^{1/2}$. | (S8) |
| --- | --- | --- |

Langevin fits for each data set are displayed in Figure S2 and the magnetic diameters are given in Table S6.

Table S6. Magnetic diameters from Langevin fitting procedure.

| **Sample** | **Physical Core Diameter (nm)** | **Magnetic Diameter**  **(nm)** |
| --- | --- | --- |
| **A** | 4.9 ± 0.7 | 5.4 ± 0.04 |
| **B** | 8.5 ± 0.9 | 9.1 ± 0.1 |
| **C** | 12.9 ± 1.1 | 11.3 ± 0.2 |
| **D** | 15.7 ± 1.5 | 11.1 ± 0.2 |
| **Ferumoxytol** | --- | 6.9 ± 0.09 |
| **Ferumoxides** | --- | 8.0 ± 0.1 |


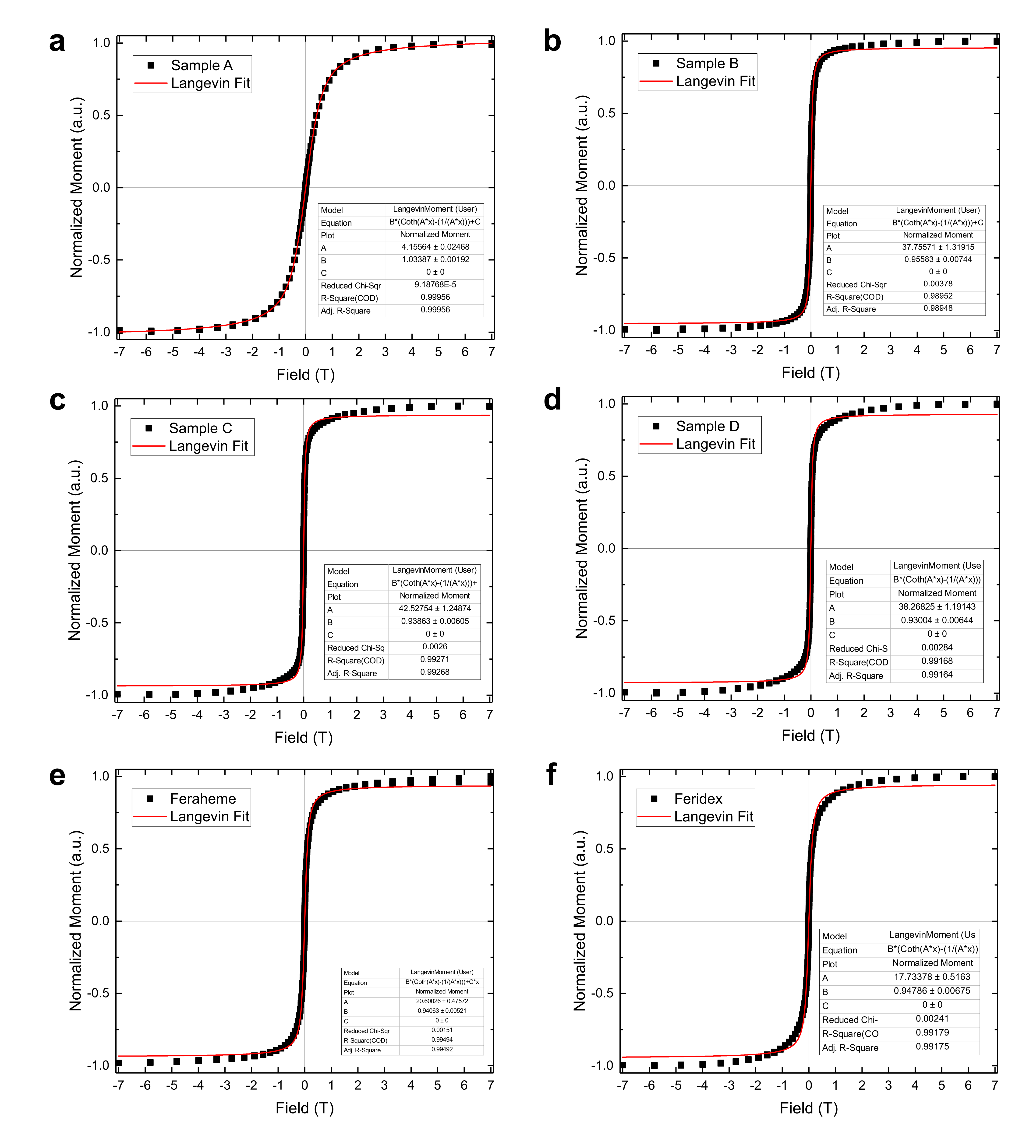


Figure S2: Langevin fits for each of the SPION-based contrast agents: (a) Sample A (4.9 nm), (b) Sample B (8.5 nm), (c) Sample C (12.9 nm), (d) Sample D (15.7 nm), (e) ferumoxytol, and (f) ferumoxides.

**Magnetization measurements of Gd-BOPTA.** The magnetization of Multihance (Gd-BOPTA) was measured by subtracting the magnetization of two samples: (1) 80 μL of 10 mmol/L Gd-BOPTA diluted in deionized water and (2) 80 μL of deionized water. The magnetization of both samples was measured in a liquid phase at room temperature (21.5 °C). Each sample was measured using the same polypropylene vial so that background magnetization would be the same. The magnetization of Gd-BOPTA alone was calculated by subtracting the magnetization of the deionized water from the dilute Gd-BOPTA sample.

E. General Inversion Recovery Equation for Fits in Figure 3 of Main Paper

Solid lines in Figure 3 (main paper) were generated by fitting the data to a general inversion recovery equation of the form *S* = *S_o_* (1 - 2*e^-TI/T1^* + *e^-TR/T1^*), where *S* is the signal, TI is the inversion time, TR is the repetition time, and T1 is the longitudinal relaxation time. This equation assumes a perfect inversion, differing from Equation 2 in the main paper, which has a factor to account for an imperfect inversion in the T_1_ mapping.

F. Relaxivity Analysis

**Slice Selection.** At 64 mT, two slices at either end of the sample region were removed from analysis to avoid artifacts from the ends of the sample tubes. The slice thickness was 5 mm, so this removed at most 10 mm from the top and bottom of the samples. The 50 mL centrifuge tubes used to prepare the samples had a length of 117.5 mm from base to cap and were filled almost entirely; so, most of the sample volume remained after removing slices. At 3 T, a single slice from either end of the sample region was removed from analysis to avoid artifacts from the end of the samples. Slice thickness was 2 mm with a 4 mm gap between slices, so this removed at most 6 mm from the top and bottom of samples.

**Data Selection.** For some of the contrast agents, the highest concentrations caused MRI signal to decay more quickly than the first inversion time at 64 mT. For these cases, T_1_ could not be measured accurately, since the signal had exponentially relaxed before the first point of measurement. So, we instituted a selection rule to exclude cases where the concentration of contrast agents was too high for accurate determination of measurement times. The rule states that if the T_1_ value calculated using a fit of equation (2) (in main paper) is less than the first inversion time (100 ms for 64 mT), then the data was excluded from the relaxivity fit. An example of this exclusion rule applied to Sample D (15.7 nm SPIONs) is shown below.

Table S7. Parameters for example of data exclusion rule corresponding to Sample D.

| **Nominal Concentration (mmol/L)** | **Actual Concentration, ICP-OES (mmol/L)** | **Mean T_1_ Across Slices (ms)** | **Excluded because Mean T_1_ < 100 ms** |
| --- | --- | --- | --- |
| 0 | 0 | 1621.14 | No |
| 0.03 | 0.034 | 376.09 | No |
| 0.06 | 0.069 | 266.97 | No |
| 0.12 | 0.14 | 100.72 | No |
| 0.25 | 0.29 | 50.84 | Yes |
| 0.5 | 0.57 | 39.98 | Yes |
| 1 | 1.15 | 38.15 | Yes |


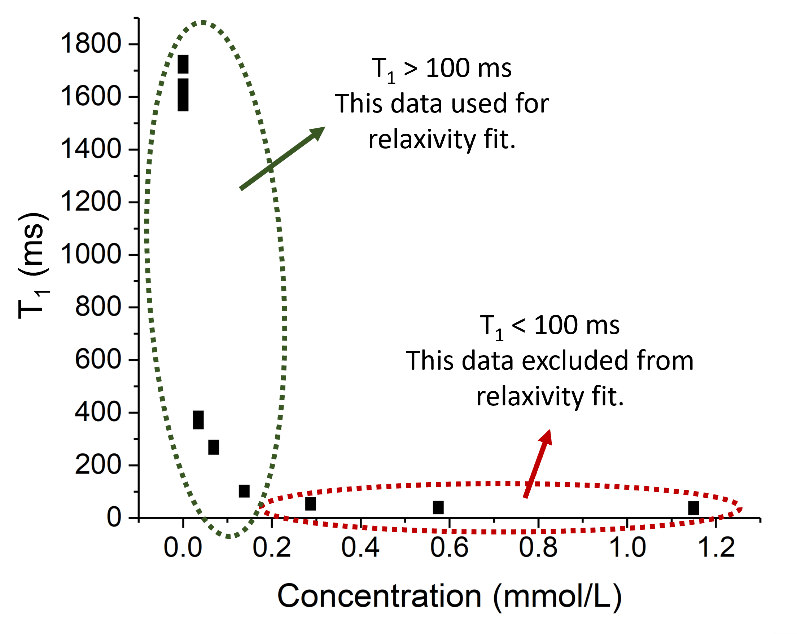


Figure S3: T_1_ times as a function of concentration for Sample D (15.7 nm SPIONs). Data points within the dashed green oval were selected for relaxivity fitting. Data in the dashed red oval were not included because T_1_ was measured to be less than the first inversion time (100 ms) of the inversion recovery pulse sequence.

Our justification for use of this selection rule is clear when the relaxation rate (R_1_= 1/T_1_) is plotted as a function of concentration. R_1_ (1/T_1_) should exhibit a linear relationship as a function of concentration. As Figure S4 shows, the R_1_ (1/T_1_) values excluded from relaxivity analysis do not follow this trend because they could not be measured accurately by the inversion recovery sequence.


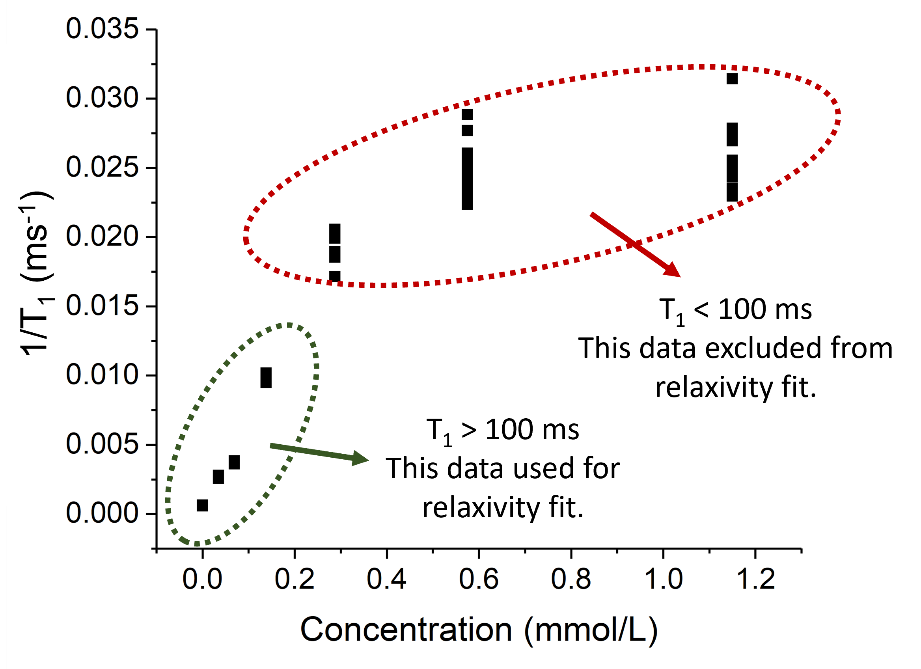


Figure S4: T_1_ relaxation rate as a function of concentration for Sample D (15.7 nm SPIONs). Data points within the dashed green oval were selected for relaxivity fitting. Data in the dashed red oval were not included because T_1_ was measured to be less than the first inversion time (100 ms) of the inversion recovery pulse sequence. Note that the excluded data (dashed red oval) do not follow the expected linear relationship between T_1_ relaxation rate and concentration.

Table S8. List of data excluded for each sample based on the “T_1_ < 100 ms” selection rule described above.

|  | **Sample A** | **Sample B** | **Sample C** | **Sample D** | **Ferumoxytol** | **Ferumoxides** | **Gd-BOPTA** |
| --- | --- | --- | --- | --- | --- | --- | --- |
| **0 mmol/L** | ✔ | ✔ | ✔ | ✔ | ✔ | ✔ | ✔ |
| **0.06 mmol/L** | ✔ | ✔ | ✔ | ✔ | ✔ | ✔ | ✔ |
| **0.12 mmol/L** | ✔ | ✔ | ✔ | ✔ | ✔ | ✔ | ✔ |
| **0.25 mmol/L** | ✔ | ✔ | **✖** | **✖** | ✔ | ✔ | ✔ |
| **0.5 mmol/L** | ✔ | ✔ | **✖** | **✖** | **✖** | **✖** | ✔ |
| **1 mmol/L** | ✔ | **✖** | **✖** | **✖** | **✖** | **✖** | ✔ |

**Linear Fitting for Relaxivity Determination.** After the selection rule was applied, the remaining data was fit using a linear regression with OriginPro 2017. The slope coefficients were recorded and reported in the main text as the r_1_ and r_2_ relaxivities. Plots of the fits for 64 mT and 3 T are displayed below.


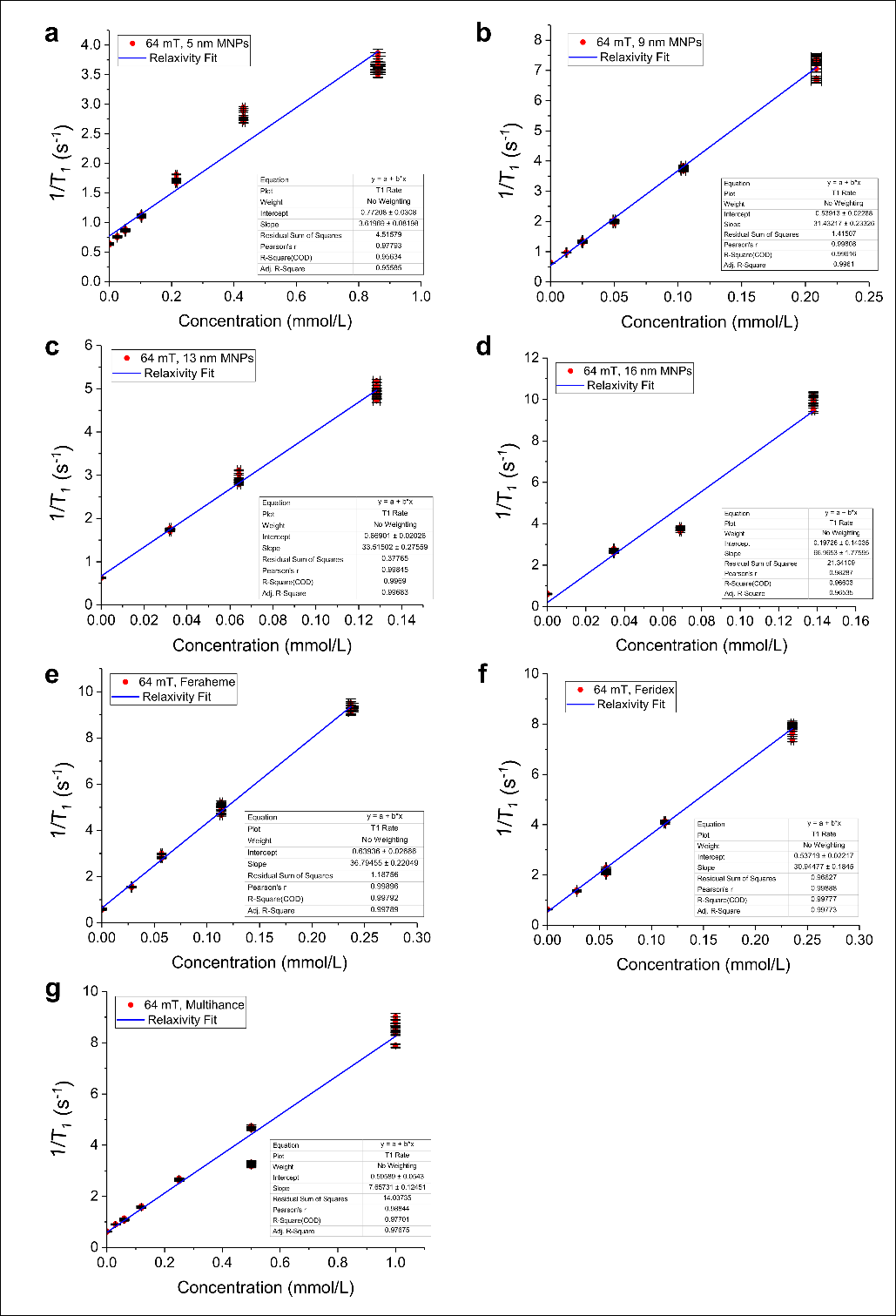


Figure S5: Longitudinal relaxivity fits for each contrast agents at 64 mT: (a) Sample A (4.9 nm), (b) Sample B (8.5 nm), (c) Sample C (12.9 nm), (d) Sample D (15.7 nm), (e) ferumoxytol, (f) ferumoxides, and (g) Gd-BOPTA.

**
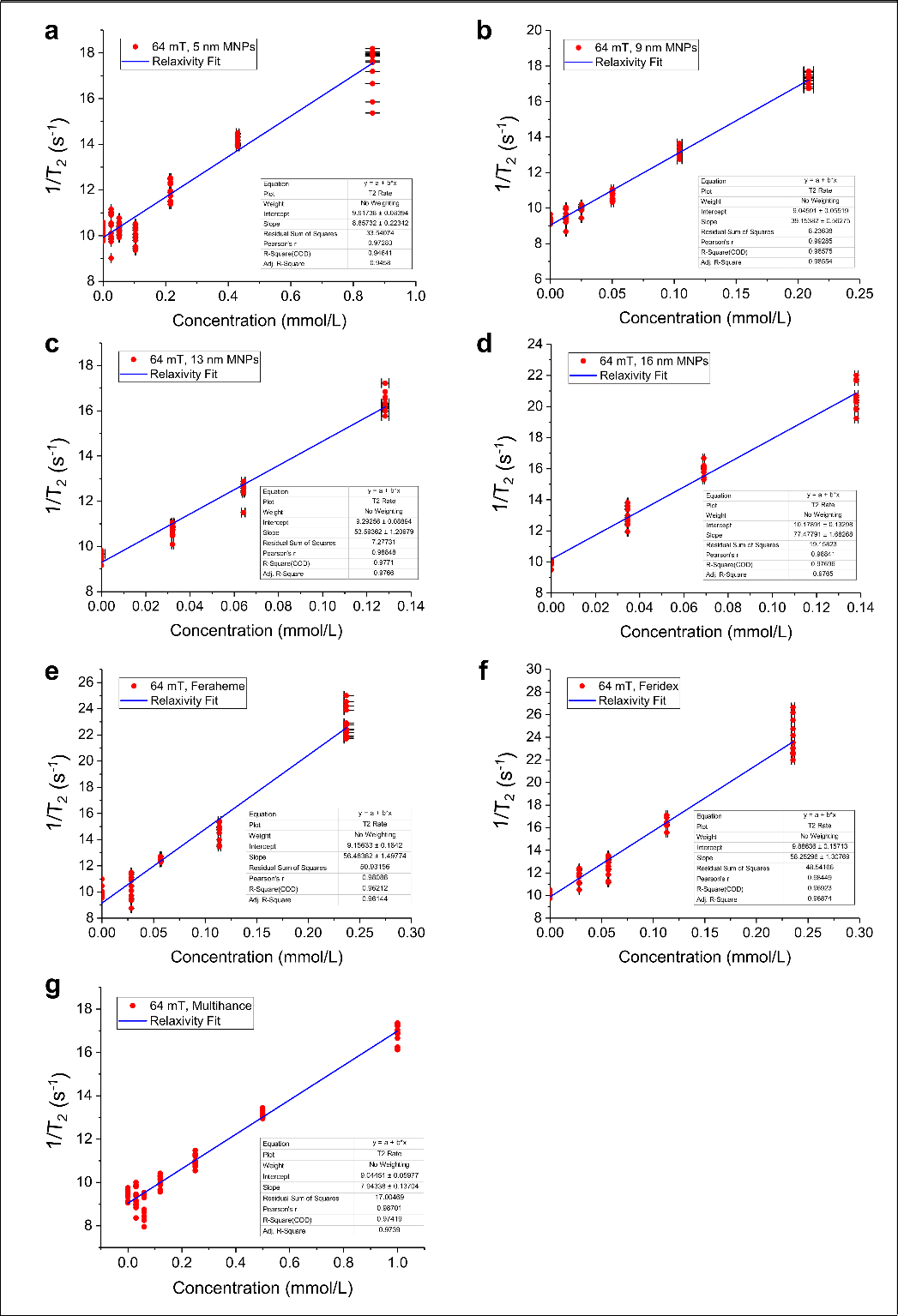
**

Figure S6: Transverse relaxivity fits for each contrast agents at 64 mT: (a) Sample A (4.9 nm), (b) Sample B (8.5 nm), (c) Sample C (12.9 nm), (d) Sample D (15.7 nm), (e) ferumoxytol, (f) ferumoxides, and (g) Gd-BOPTA.

**
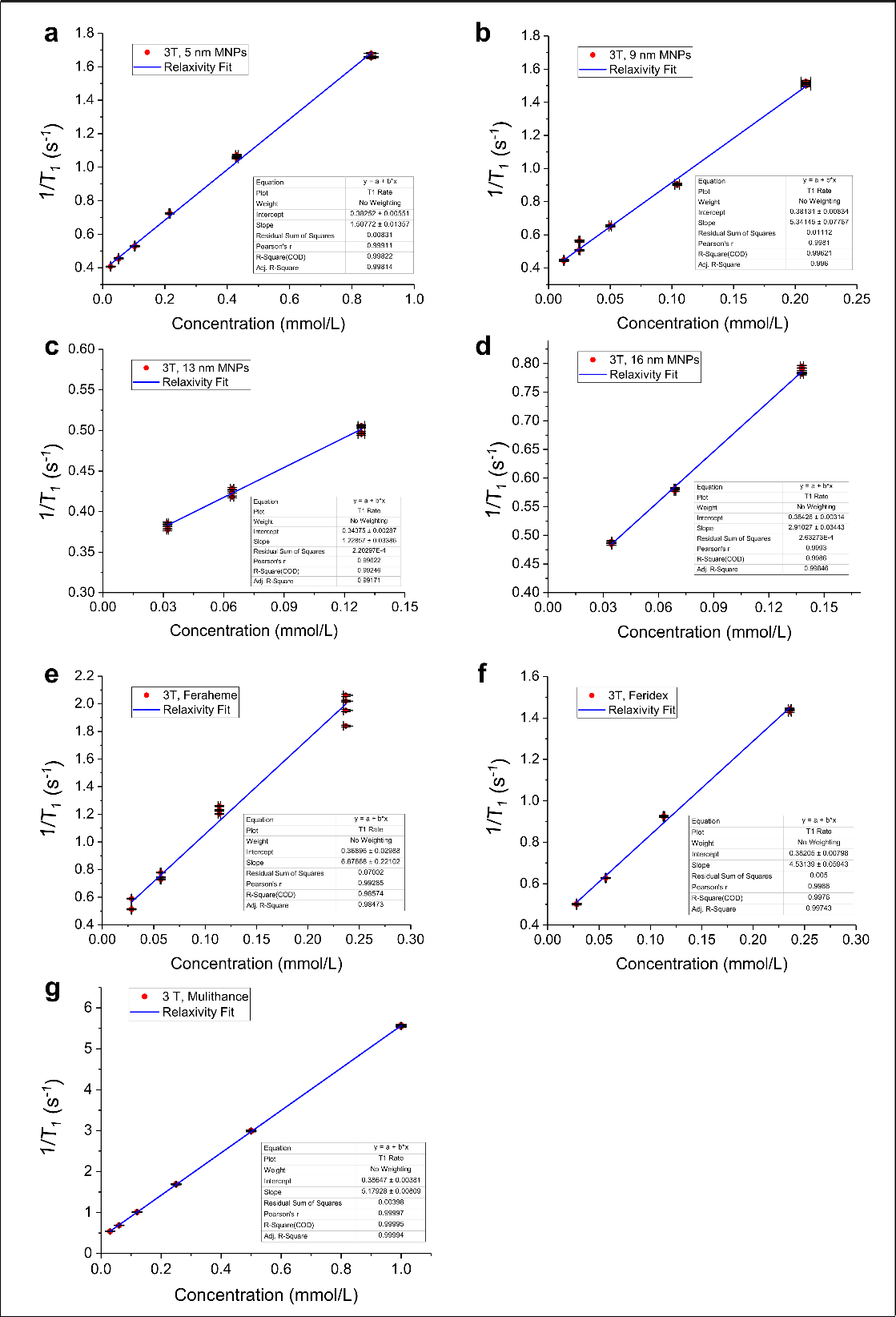
**

Figure S7: Longitudinal relaxivity fits for each contrast agents at 3 T: (a) Sample A (4.9 nm), (b) Sample B (8.5 nm), (c) Sample C (12.9 nm), (d) Sample D (15.7 nm), (e) ferumoxytol, (f) ferumoxides, and (g) Gd-BOPTA.

**
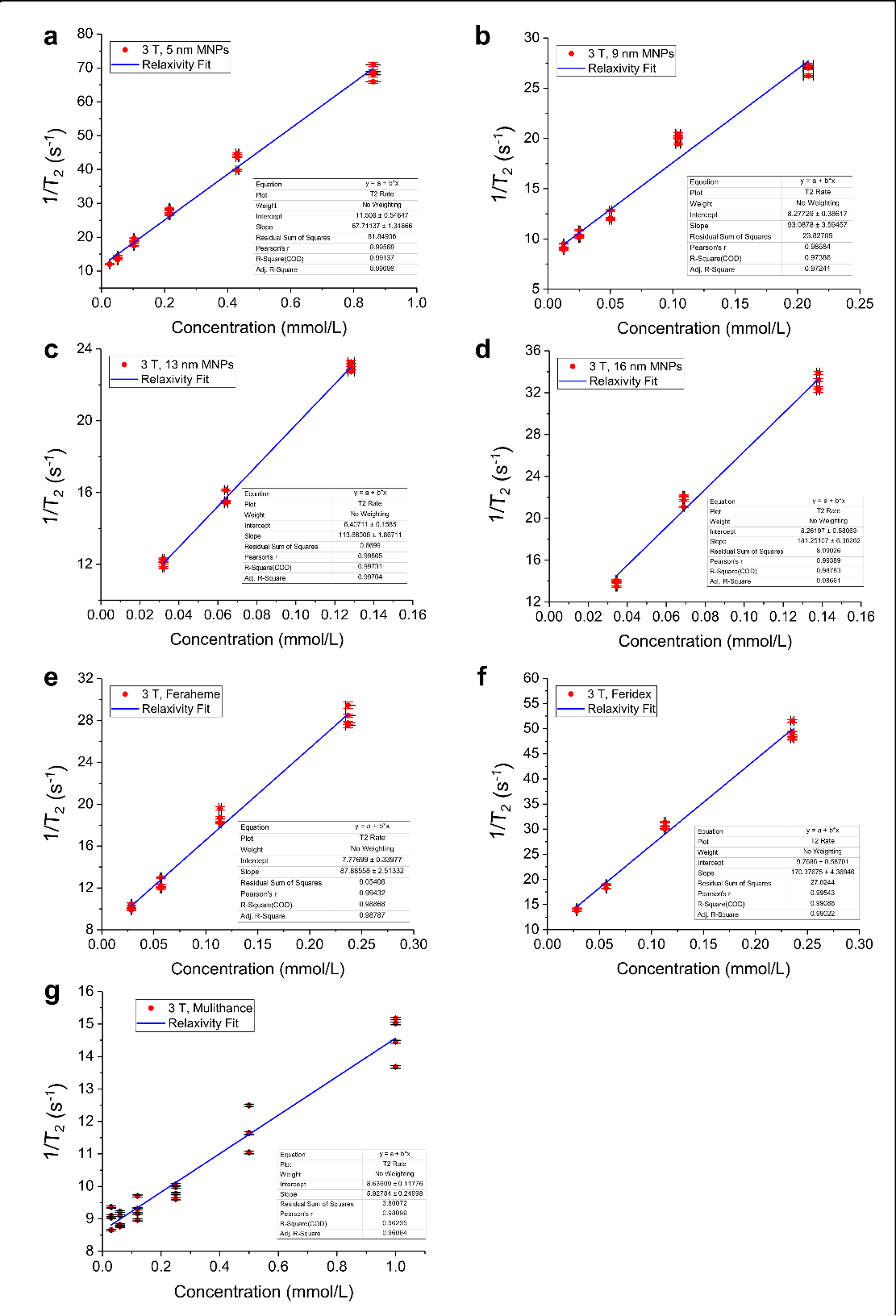
**

Figure S8: Transverse relaxivity fits for each contrast agents at 3 T: (a) Sample A (4.9 nm), (b) Sample B (8.5 nm), (c) Sample C (12.9 nm), (d) Sample D (15.7 nm), (e) ferumoxytol, (f) ferumoxides, and (g) Gd-BOPTA.

**
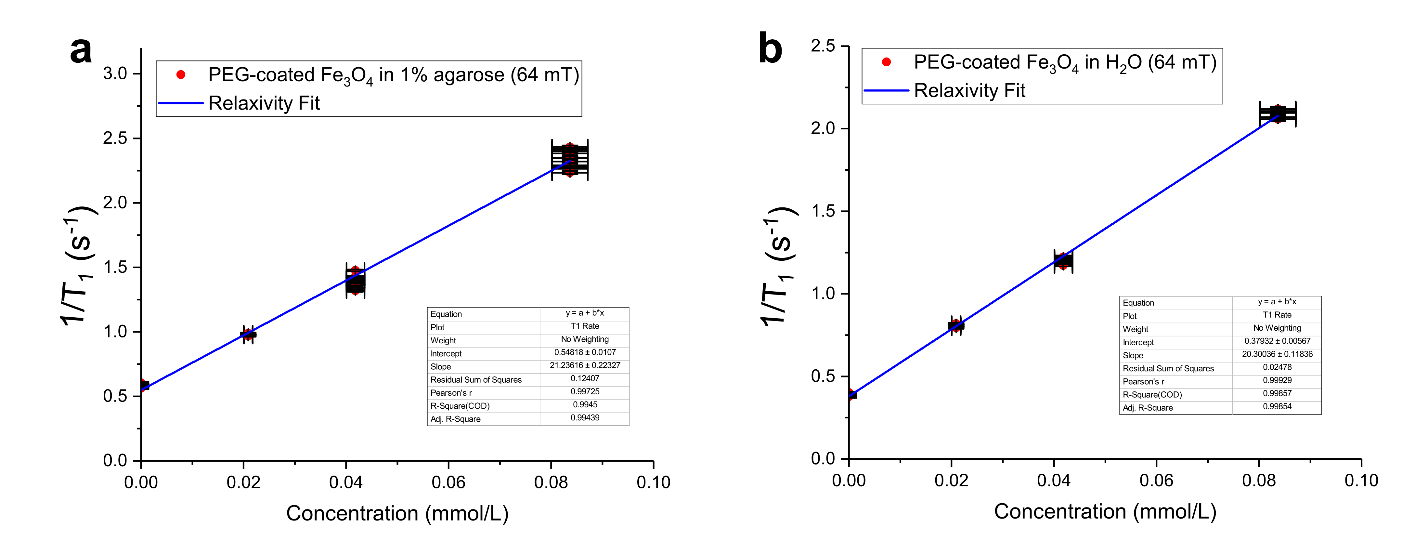
**

Figure S9: Longitudinal relaxivity fits for PEG-coated Fe_3_O_4_ at 64 mT suspended in (a) agarose and (b) deionized H_2_O.

**
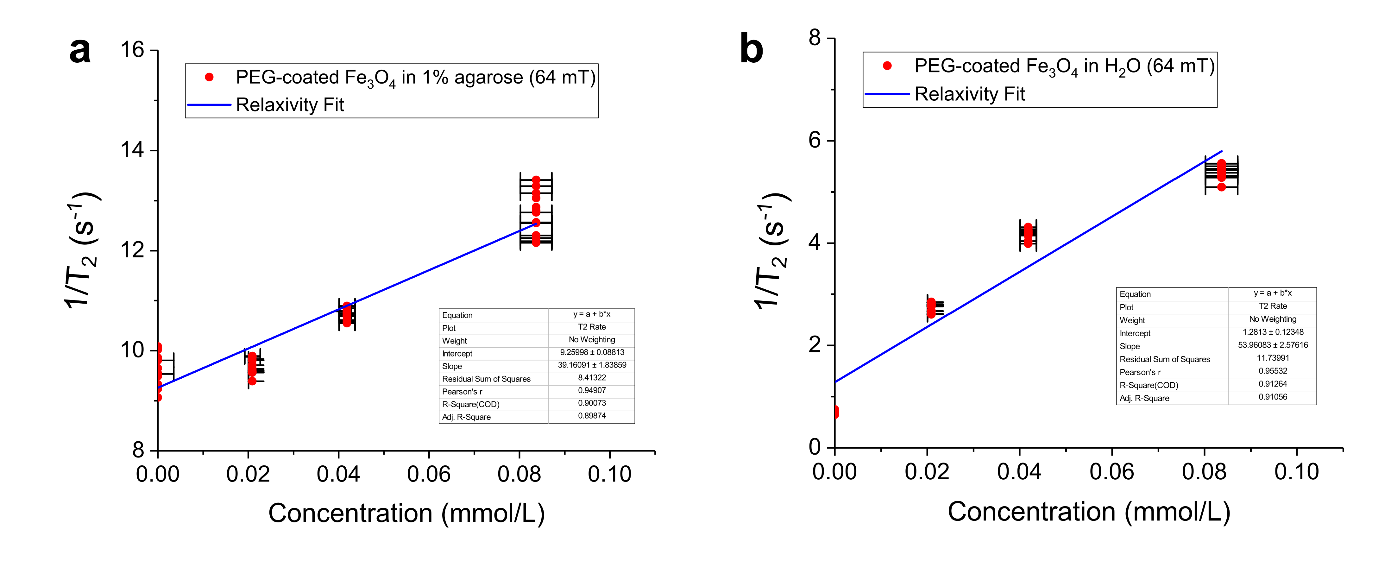
**

Figure S10: Transverse relaxivity fits for PEG-coated Fe_3_O_4_ at 64 mT suspended in (a) agarose and (b) deionized H_2_O.

G. Nuclear Magnetic Resonance Dispersion (NMRD) Details

**NMRD Data of Agarose Gel.** Figure S11 shows the longitudinal relaxation rates of the 1 % mass fraction agarose gel in water as a function of proton Larmor frequency. The data was acquired using NMRD measurements. The sample was measured at 15 °C, 21.5 °C, 25 °C, and 37 °C. These curves were subtracted from the NMRD data for each contrast agent to separate the relaxation of the contrast agents and the agarose medium.


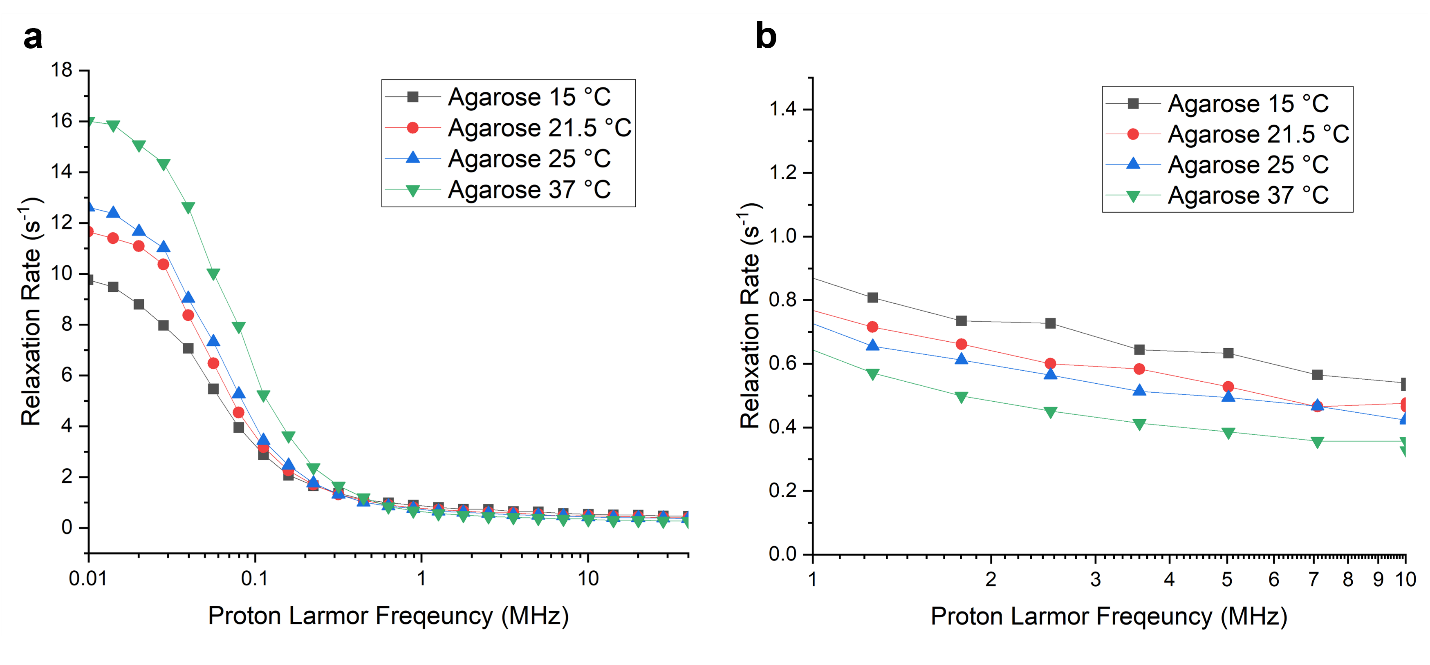


Figure S11: NMRD measurements on 1 % mass fraction agarose gel in water. (a) Longitudinal relaxation rate as a function of proton Larmor frequency for 15 °C, 21.5 °C, 25 °C, and 37 °C. (b) Magnified domain from 1 MHz to 10 MHz showing agarose relaxation rate in the range of the 64 mT operating frequency.

The data in Figure S11a shows variation in the relaxation rate at low frequency for each temperature condition. At about 0.7 MHz, however, the relaxation rate at each temperature drops to the order of 1 s^-1^. Figure S11b shows a magnified frequency domain from 1 MHz to 10 Mhz, which contains the operating frequency of the 64 mT scanner (2.7 MHz). Within this frequency range, the relaxation rate from agarose is low compared to the SPION-based contrast agents. Therefore, the agarose relaxation did not contribute significantly to relaxation-based measurements at the 64 mT operating frequency.

In general, agarose begins to melt around 40 °C; although, we observed no appreciable phase transition type effects at the highest temperature of 37 °C. Still, we tried to account for possible changes due to melting by (1) subtracting the agarose data away from the NMRD contrast agent data at each temperature and (2) by fitting the NMRD data at 37 °C with the diffusion coefficient, *D*, as a free fitting parameter (details in “NMRD Fitting for SPION-Based Agents”).

**NMRD Data of Contrast Agents.** ^1^H NMRD data for all contrast agents at 15 °C, 21.5 °C, 25 °C and 37 °C are displayed in Figure S12.


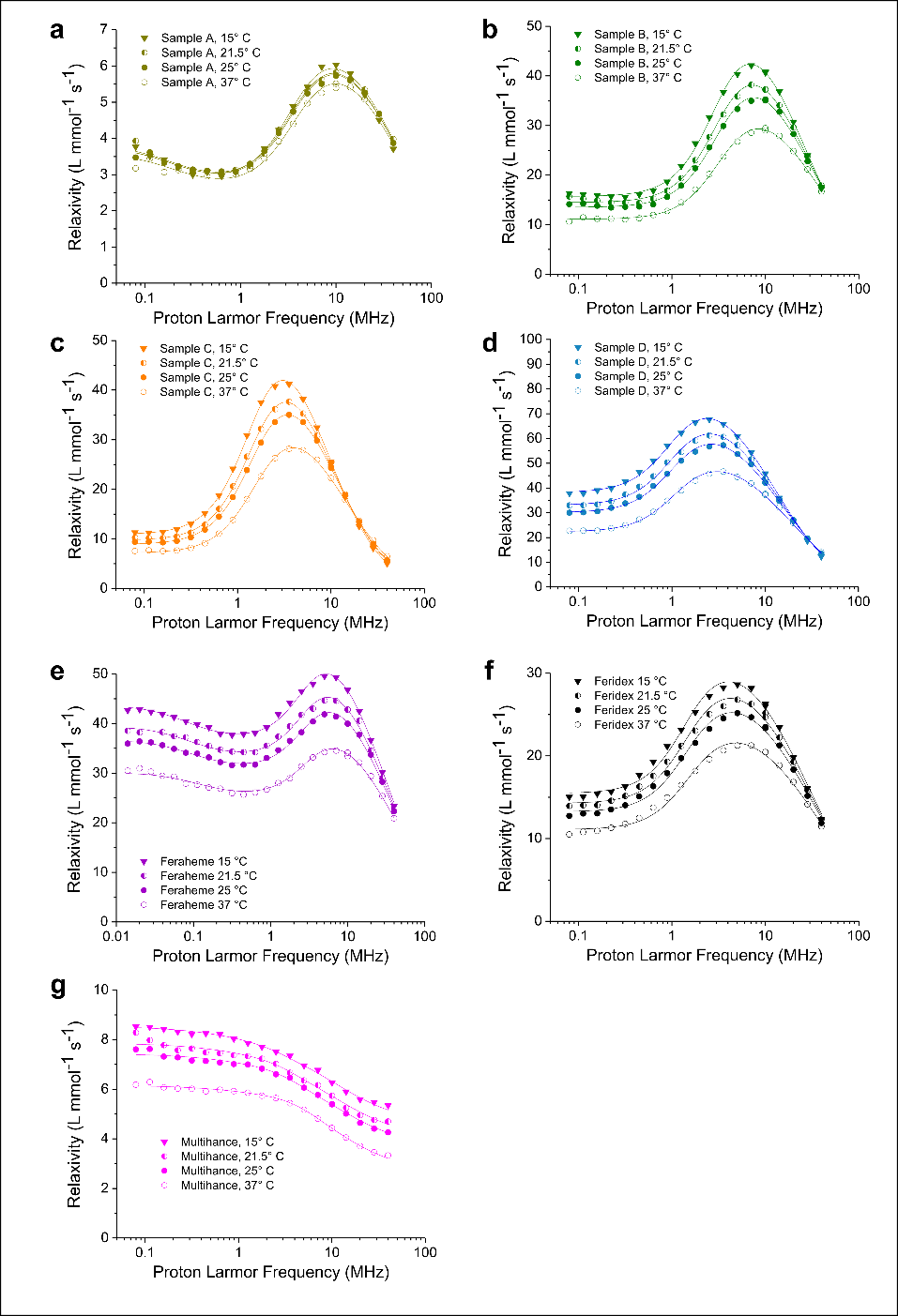


Figure S12: NMRD curves showing longitudinal relaxivity as a function of proton Larmor frequency and corresponding theoretical fits for (a) Sample A (4.9 nm), (b) Sample B (8.5 nm), (c) Sample C (12.9 nm), (d) Sample D (15.7 nm), (e) ferumoxytol, (f) ferumoxides, and (g) Gd-BOPTA.

**NMRD Fitting for SPION-Based Agents.** The profiles were fit using the following equation^2,3^

|  | $R_{1}^{\mathrm{out}}=\frac{32\pi}{135}\left( \frac{\mu_{0}}{4\pi} \right)^{2}\frac{{1000N_{A}\left[ M \right]\left( \gamma_{I}\mu_{B}g_{e} \right)}^{2}S^{2}}{dD}\left\{ 3\left[ L(x) \right]^{2}J^{A}\left( \omega_{I}, \tau_{D} \right)+\left[ 3\left( 1-2\frac{L\left( x \right)}{x}-\left[ L(x) \right]^{2} \right)+7Q\frac{L\left( x \right)}{x} \right]J^{F}\left( \omega_{I},\tau_{D},\tau_{e} \right)+7P\frac{L\left( x \right)}{x}J^{F}\left( \omega_{0}, \tau_{D},\tau_{e} \right) \right\},$ | (S9) |
| --- | --- | --- |

where the fit parameters are (i) the distance of closest approach, *d*, (ii) the single large spin, *S*, resulting from the coupling of all electron spins, (iii) the Néel correlation time, $\tau_{e}$, (iv) the molar concentration of the NPs, [*M*], in mol/L, (v) the diffusion coefficient, *D*, and the heuristic parameters *P* and *Q.* These parameters account for anisotropic effects at low field, such as those generated by the orbital magnetic moments, which cause the average magnetic moment to not align along the direction of the applied magnetic field. In equation (S9), *N*_A_ is Avagadro’s number, $\omega_{S}=\mu_{B}g_{e}B_{0}\boldsymbol{/}\hbar$,$x=\frac{S\hbar\omega_{S}}{kT}$, $L\left( x \right)=\coth x-\frac{1}{x}$ , and $\tau_{D}=\frac{d^{2}}{D}$ . The spectral density functions in equation (S9) are

|  | $J^{A}\left( \omega, \tau_{D} \right)=\frac{1+\frac{5z}{8}+\frac{z^{2}}{8}}{1+z+\frac{z^{2}}{2}+\frac{z^{3}}{6}+\frac{4z^{4}}{81}+\frac{z^{5}}{81}+\frac{z^{6}}{648}}; z=\left( 2\omega\tau_{D} \right)^{1/2},$ | (S10) |
| --- | --- | --- |

and

|  | $J^{F}\left( \omega, \tau_{D},\tau_{e} \right)=\mathrm{Re}\left[ \frac{1+\frac{\Omega^{1/2}}{4}}{1+\Omega^{1/2}+\frac{4\Omega}{9}+\frac{\Omega^{3/2}}{9}} \right]; \Omega=i\omega\tau_{D}+\frac{\tau_{D}}{\tau_{e}}.$ | (S11) |
| --- | --- | --- |

In the case of an anisotropy energy much larger than the Zeeman energy ($\mu_{B}g_{e}B_{0}$), *P* = 0 and *Q* = 1, which corresponds to a vanishing of the low field dispersion before the peak in relaxivity. In this case, the particle’s magnetization is fixed along the easy axis of magnetization, which is also parallel to B­_0_. This case usually occurs for relatively large NPs, and the validity of this limit is experimentally shown by the absence of low field dispersions.

The data acquired at all temperatures were fitted simultaneously with common values of *d*, *S*, [*M*], and with a fixed value of *D* at 21.5 °C. The values of $\tau_{e}$ at all temperatures and of *D* at 15 °C, 25 °C and 37 °C were allowed to change. *P* was fixed to 0 and *Q* to 1 for all profiles except those of the Sample A and for ferumoxytol, in which a low field dispersion seems present. In this case *P* was varied as a fit parameter and *Q* was set to 1− *P*.

Table S9: Best fit parameters for SPION-based samples. A bold value indicates that it was fixed for the entire column.

|  | [NP]  (mmol/L) | S | d  (nm) | D^15 °C^  (10^-9^ m^2^/s) | τ_e_^15 °C^  (10^-9^ s) | D^21 °C^  (10^-9^ m^2^/s) | τ_e_ ^21 °C^  (10^-9^ s) | D^25 °C^  (10^-9^ m^2^/s) | τ_e_ ^25 °C^  (10^-9^ s) | D^37 °C^  (10^-9^ m^2^/s) | τ_e_ ^37 °C^  (10^-9^ s) |
| --- | --- | --- | --- | --- | --- | --- | --- | --- | --- | --- | --- |
| Sample A^(d)^ | 6.75 × 10^-6^ | 6160 | 3.9 | 1.94 | 2.51 | **2** | 2.66 | 2.01 | 2.60 | 2.10 | 2.46 |
| Sample B | 6.83 × 10^-5^ | 6730 | 5.7 | 1.71 | 1.72 |  | 1.63 | 2.20 | 1.54 | 2.86 | 1.30 |
| Sample C | 3.67 × 10^-5^ | 13700 | 10.3 | 1.67 | 2.65 |  | 2.38 | 2.23 | 2.19 | 3.06 | 1.81 |
| Sample D | 1.19 × 10^-5^ | 20700 | 7.4 | 1.80 | 8.09 |  | 6.80 | 2.16 | 6.10 | 2.76 | 4.36 |
| Ferumoxides | 5.39 × 10^-6^ | 15100 | 4.7 | 1.84 | 3.58 |  | 3.30 | 2.16 | 3.07 | 2.58 | 2.58 |
| Ferumoxytol^(e)^ | 3.74 × 10^-5^ | 7600 | 4.6 | 1.77 | 9.04 |  | 8.43 | 2.18 | 8.00 | 2.73 | 6.95 |

(d) P=0.36

(e) P=0.22

If *D* is constrained to be the same for all the samples at the same temperature, equally good fits can be obtained by allowing *S* to vary by within 10 % for different temperature conditions.

**NMRD Fitting for Gd-BOPTA.** The relaxivity, *r*_1_, is defined as the paramagnetic enhancement of the solvent nuclear relaxation rate in the presence of 0.001 mol/L of paramagnetic metal ions in solution and is given by

|  | $r_{1}=\frac{0.001q}{55.6}\left( R_{1M}^{-1}+\tau_{M} \right)^{-1}+r_{1\mathrm{OS}},$ | (S12) |
| --- | --- | --- |

where *q* is the number of water molecules coordinated to the paramagnetic ion, *τ*_M_ is their exchange time, and $r_{1\mathrm{OS}}$ is the outer-sphere relaxivity (see below). *R*_1_*_M_* is the paramagnetic relaxation rate due to the point dipole-point dipole interaction between the magnetic moments of the water protons coordinated to the gadolinium ion and the magnetic moment of the unpaired electron(s) of the gadolinium ion, and is described by the Solomon equation

|  | $R_{1M}=\frac{2}{15}\left( \frac{\mu_{0}}{4\pi} \right)^{2}\frac{\gamma_{I}^{2}g_{e}^{2}\mu_{B}^{2}S(S+1)}{r^{6}}\left( \frac{7\tau_{c}}{1+\omega_{S}^{2}\tau_{c}^{2}}+\frac{3\tau_{c}}{1+\omega_{I}^{2}\tau_{c}^{2}} \right),$ | (S13) |
| --- | --- | --- |

where $\mu_{0}$ is the permeability of the vacuum, $\gamma_{I}$ is the proton magnetogyric ratio, $\mu_{B}$ is the electron Bohr magneton, *S* is the electron spin quantum number, *r* is the distance between paramagnetic metal and protons of the coordinated water molecule, $\omega_{S}$ and $\omega_{I}$ are the electron and proton Larmor frequency, respectively, and the correlation time $\tau_{c}$ is given by

|  | $\tau_{c}^{-1}= \tau_{R}^{-1}+\tau_{M}^{-1}+R_{1e},$ | (S14) |
| --- | --- | --- |

where $\tau_{R}$ is the reorientation correlation time of the gadolinium complex, and $R_{1e}$ is the electron relaxation rate. Using the pseudorotation model, $R_{1e}$ is described by the Bloembergen-Morgan equation

|  | $R_{1e}=\frac{2\Delta_{t}^{2}}{50}\left[ 4S\left( S+1 \right)-3 \right]\left( \frac{\tau_{v}}{1+\omega_{S}^{2}\tau_{v}^{2}}+\frac{4\tau_{v}}{1+4\omega_{S}^{2}\tau_{v}^{2}} \right),$ | (S15) |
| --- | --- | --- |

where $\Delta_{t}^{2}$ is the mean squared fluctuation of the zero-field splitting (ZFS), called squared transient ZFS, and $\tau_{v}$ is the correlation time for the instantaneous distortions of the metal coordination polyhedron.

The outer-sphere relaxivity is due to the water molecules diffusing around the paramagnetic metal ion, and according to the commonly used force-free hard sphere model, it is given by

|  | $r_{1\mathrm{OS}}=\frac{32\pi}{405}\left( \frac{\mu_{0}}{4\pi} \right)^{2}\frac{N_{A}\gamma_{I}^{2}g_{e}^{2}\mu_{B}^{2}S(S+1)}{dD}\left( 7J^{\mathrm{tr}}\left( \omega_{S} \right)+3J^{\mathrm{tr}}\left( \omega_{I} \right) \right),$ | (S16) |
| --- | --- | --- |

where $N_{A}$ is the Avogadro constant, *d* is the distance of closest approach between the ligand nuclei and the paramagnetic metal ion, *D* is the sum of the diffusion coefficients of the paramagnetic ion (or complex ion) and of the ligand molecule, and

|  | $J^{\mathrm{tr}}\left( \omega\right)=\frac{1+\frac{5z}{8}+\frac{z^{2}}{8}}{1+z+\frac{z^{2}}{2}+\frac{z^{3}}{6}+\frac{4z^{4}}{81}+\frac{z^{5}}{81}+\frac{z^{6}}{648}};$ | (S17) |
| --- | --- | --- |
|  | $z=\sqrt{2\left( \omega\tau_{D}+R_{1e}\tau_{D} \right)};$ | (S18) |
|  | $\tau_{D}=\frac{d^{2}}{D}$. | (S19) |

Table S10. Best fit parameters of Gd-BOPTA:

|  | **15 °C** | **21.5 °C** | **25 °C** | **37 °C** |
| --- | --- | --- | --- | --- |
| $\Delta_{t}$ (cm^-1^) | 0.0188 | | | |
| $\tau_{v}$ (ps) | 41 | 40 | 39 | 36 |
| $\tau_{M}$ (μs) | 6.1 | 5.6 | 5.4 | 4.7 |
| $\tau_{R}$ (ps) | 237 | 147 | 116 | 52 |
| *r* (nm) | 0.305^a^ | | | |
| *q* | 1^a^ | | | |
| *d* (nm) | 0.36^a^ | | | |
| *D* (10^-9^ m^2^ s^-1^) | 1.7 | 2.0^a^ | 2.2 | 3.0 |

^a^parameters fixed in the analysis.

References for proton relaxation theory^4,5,6^ are listed at the end of the supplemental information.

H. Colloidal Stability of Contrast Agent Samples

When diluted in deionized water, the carboxylic acid-coated nanoparticles show poor colloidal stability and crash out of solution (see Figure S13 below).


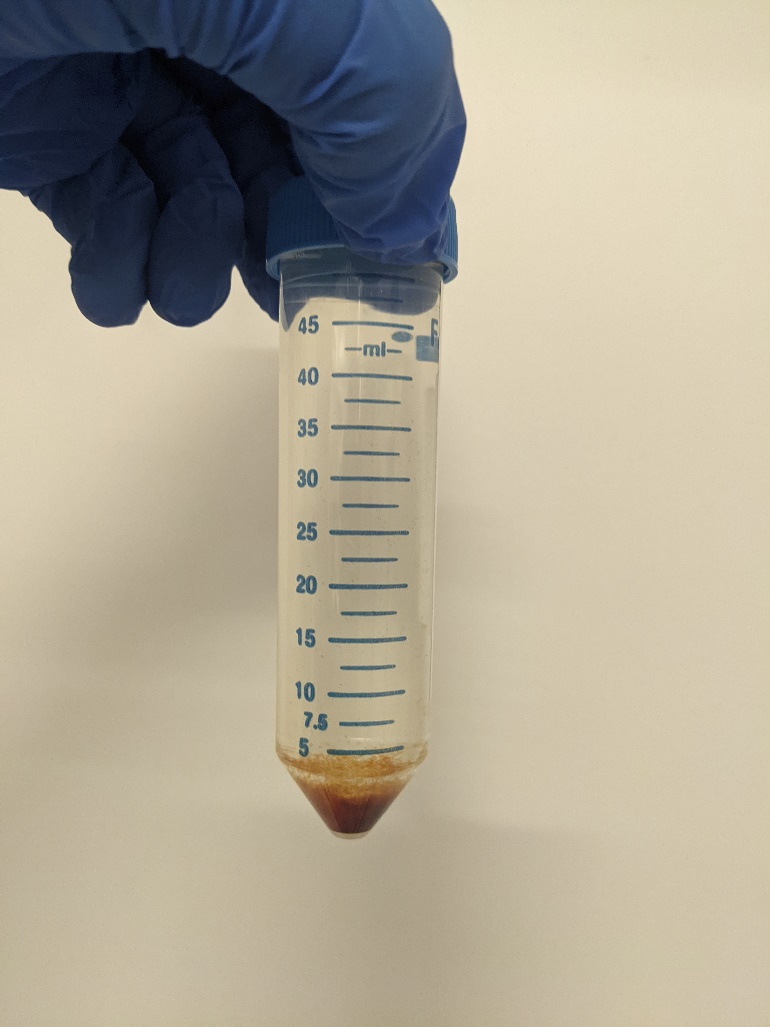


Figure S13: Carboxylic acid-coated Fe_3_O_4_ nanoparticles are not colloidally stable when diluted in deionized water. They form a reddish-brown precipitate at the bottom of a 50 mL centrifuge tube.

I. Comparison of Relaxivity in Water and Agarose Gel

To explore the effect of the agarose embedding gel, we measured the relaxivities of 11.8 nm ± 1.01 nm PEG-coated SPIONs in both deionized water and an agarose gel (prepared with mass fraction of 1 % agarose in H_2_O). The PEG-coated SPIONs showed a much higher degree of colloidal stability in deionized water compared the carboxylic acid-coated particles. Figure S14 shows r_1_, r_2_, and r_2_/r_1_ for the deionized water environment and the agarose gel. The longitudinal relaxivities differ by less than 5 %. The r_2_ value increases in the deionized water by 38 %, which may be related to differences between aggregation of PEGylated particles in H_2_O versus agarose.


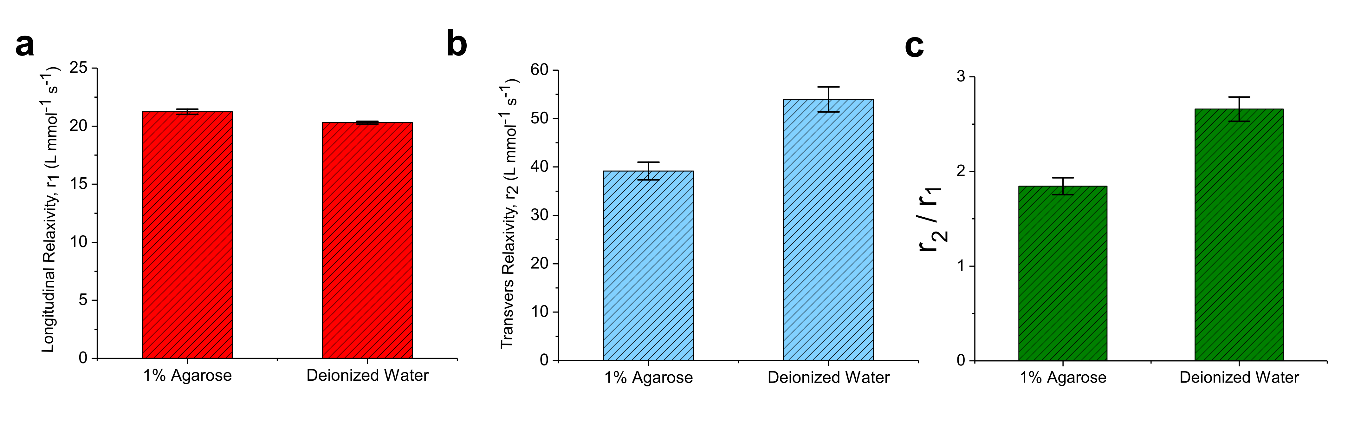


Figure S14: Comparison of (a) longitudinal relaxivity, *r_1_,* (b) transverse relaxivity, *r_2_*, and (c) ratio *r_2_/r_1_* for 11.8 nm PEG-coated SPIONs in agarose and 11.8 nm PEG-coated SPIONs deionized water.

J. Calculation of Relaxivity Curves with Diffusion Coefficients Corresponding to Different Bio-Environments Using Relaxation Model from NMRD Analysis

We investigated the relationship between longitudinal relaxivity and particle environment for ferumoxytol by calculating NMRD curves using diffusion coefficients for various types of bio-fluids and tissues. The NMRD curves were calculated using the model proposed by Roch et al.^2^, which is also the same model used to fit the NMRD data. To calculate the curves, we held all parameters constant for the fit to experimental data of ferumoxytol in agarose at 37 °C except for the diffusion coefficient, which was changed to match literature values for bio-fluids and tissue. We used diffusion coefficients corresponding to different bio-fluids and tissues that are found in the brain since the 64 mT scanner is currently FDA-approved for neuroimaging. The bio-fluids and tissues include blood, cerebrospinal fluid, white matter, grey matter, and water. We note that the relationship between longitudinal relaxivity for SPION-based contrast agents and the diffusion coefficients of surrounding tissue is more complicated than the prediction here. Diffusion of protons in the vicinity of particle cores will be influenced by particle surface coatings as well as diffusion of protons throughout tissue. Ferumoxytol, for example, has iron oxide cores embedded in a polymer matrix, which will affect proton diffusion. Therefore, the calculation here reflects an idealized situation where isolated ferumoxytol cores are dispersed in an environment with a uniform diffusion coefficient. Still, the predictions show some interesting trends, such as an increased longitudinal relaxivity with decreased diffusion coefficient.


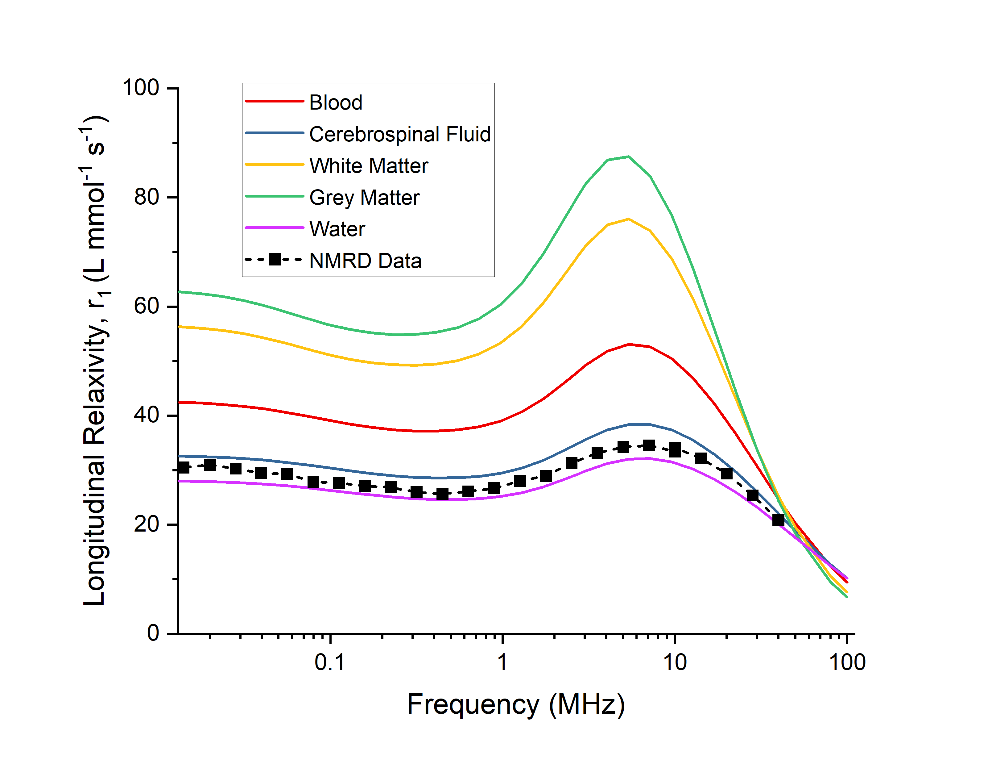


Figure S15: Theoretical prediction of longitudinal relaxivity, *r_1_*, for ferumoxytol cores in various types of fluids and biological tissues. The original NMRD data for ferumoxytol in agarose at 37 °C is also displayed.

Table S11. Diffusion coefficients and computed relaxivity at 64 mT for various types of fluids and tissues.

| Tissue or Fluid Type | 64 mT r_1_  (L mmol^-1^ sec^-1^) | Diffusion Coefficient  at 37 °C  (10^-9^ m^2^/s) | References |
| --- | --- | --- | --- |
| Blood | 48.0 | 1.54 | (7) |
| Cerebrospinal Fluid | 34.9 | 2.4 | (8), (9) |
| White Matter | 69.1 | 0.89 | (10) |
| Grey Matter | 80.1 | 0.7 | (10) |
| Water | 29.2 | 3.03* | (11) |

*Diffusion coefficient of water at 37 °C was estimated using linear interpolation between diffusion coefficients measured at 35 °C and 40 °C using NMR^11^.

References

1. Schneider, C. A., Rasband, W. S. & Eliceiri, K. W. NIH Image to ImageJ: 25 years of image analysis. *Nat. Methods* **9**, 671–675 (2012).

2. Roch, A., Muller, R. N. & Gillis, P. Theory of proton relaxation induced by superparamagnetic particles. *The Journal of Chemical Physics* **110**, 5403–5411 (1999).

3. Laurent, S. *et al.* Magnetic Iron Oxide Nanoparticles: Synthesis, Stabilization, Vectorization, Physicochemical Characterizations, and Biological Applications. *Chem. Rev.* **108**, 2064–2110 (2008).

4. Bloembergen, N. Proton Relaxation Times in Paramagnetic Solutions. *J. Chem. Phys.* **27**, 572–573 (1957).

5. Lauffer, R. B. Paramagnetic metal complexes as water proton relaxation agents for NMR imaging: theory and design. *Chem. Rev.* **87**, 901–927 (1987).

6. Solomon, I. Relaxation Processes in a System of Two Spins. *Phys. Rev.* **99**, 559–565 (1955).

7. Funck, C., Laun, F. B. & Wetscherek, A. Characterization of the diffusion coefficient of blood. *Magnetic Resonance in Medicine* **79**, 2752–2758 (2018).

8. Gaddamanugu, S. *et al.* Clinical applications of diffusion-weighted sequence in brain imaging: beyond stroke. *Neuroradiology* **64**, 15–30 (2022).

9. Sener, R. N. Diffusion MRI: apparent diffusion coefficient (ADC) values in the normal brain and a classification of brain disorders based on ADC values. *Computerized Medical Imaging and Graphics* **25**, 299–326 (2001).

10. Helenius, J. *et al.* Diffusion-Weighted MR Imaging in Normal Human Brains in Various Age Groups. *AJNR Am J Neuroradiol* **23**, 194–199 (2002).

11. Holz, M., Heil, S. R. & Sacco, A. Temperature-dependent self-diffusion coefficients of water and six selected molecular liquids for calibration in accurate 1H NMR PFG measurements. *Phys. Chem. Chem. Phys.* **2**, 4740–4742 (2000).
